# Emerging Nonthermal Technologies for the Production of Postbiotics

**DOI:** 10.1111/1541-4337.70335

**Published:** 2025-11-22

**Authors:** Rohit Thirumdas, Priti Mudgil

**Affiliations:** ^1^ MFPI‐Quality Control Laboratory Professor Jayashankar Telangana Agricultural University Hyderabad India; ^2^ Department of Food Science, College of Agriculture and Veterinary Medicine United Arab Emirates University Al Ain UAE

**Keywords:** functional foods, nonthermal technologies, nutraceuticals, postbiotics, probiotics, pulsed electric field, ultrasonication

## Abstract

Postbiotics, defined as nonliving microbial cells and their components that confer health benefits to the host, represent a significant advancement in functional foods and dietary supplements. Compared to probiotics and prebiotics, postbiotics offer advantages in product stability, safety, and formulation flexibility. In general practice, heat killing is a widely used method to produce postbiotics. However, heat‐killed postbiotics incur few drawbacks, such as burnt flavor, denaturation of immunomodulatory molecules, and loss of functional metabolites. This review paper examines how emerging nonthermal technologies perform compared to conventional methods in producing postbiotics, emphasizing their suitability for industrial‐scale implementation and the advantages they provide over conventional thermal processing. Based on literature, the review examines key nonthermal technologies, including high‐pressure processing (HPP), pulsed electric fields (PEFs), ultrasound, cold plasma, supercritical CO_2_ (Sc‐CO_2_), and Irradiation. Their principles and industrial applicability are examined for impact on bioactivity, stability, and functional value of postbiotics, with evaluations highlighting their strengths, limitations, and optimization potential. Recent advancements in postbiotic research and nonthermal processing indicate significant innovation opportunities. However, challenges remain in scaling up methods, refining parameters, and addressing regulatory and economic constraints. Industrial integration of nonthermal technologies requires further evidence to confirm feasibility, cost‐effectiveness, and safety compliance, identifying the key gaps in optimizing protocols for inactivation, exposure‐response relationships, and clinical impacts. The review guides readers through postbiotic fundamentals, comparison of production methods, specific nonthermal technologies, and practical implementation considerations, thereby providing a foundation for future research aimed at optimizing the use of these technologies in clinical and industrial settings.

## 1 Introduction

Consumption of adequate amounts of probiotics has shown several benefits to human health. The World Health Organization defined probiotics as living organisms beneficial to the host when consumed in the desired quantity (Joint FAO/WHO Working Group 2002). Therefore, the food/supplement must retain a minimum cell number throughout the shelf‐life to benefit the consumed host. However, there is a significant loss in the probiotic cell number during the processing, transportation, and storage requiring innovative solutions to overcome these challenges (Jankovic et al. 2010). Several research findings have supported the idea that the occurrence of health benefits is not always associated with the intake of viable probiotics. The intake of dead probiotic cells or cell components has also shown health‐promoting benefits (J. Aguilar‐Toalá et al. 2018; Pimentel et al. 2023; Scott et al. 2022; Thorakkattu et al. 2022). Based on several studies, new nomenclatures like postbiotics, paraprobiotics/postbiotics, tyndallized probiotics, pharmabiotics, psychobiotics, and probioceuticals have been introduced to the field of probiotics (Pimentel et al. 2023; Zhong et al. 2024).

In this quest recently, attention has been concentrated on postbiotics and the technological advances that facilitate their production (Guglielmetti et al. 2025; Suthar et al. 2025). International Scientific Association of Probiotics and Prebiotics (ISAPP) has defined the term postbiotics as inanimate microorganisms or their components that confer a health benefit on the host when consumed in a required dose (Salminen et al. 2021a). This broad definition significantly expands the traditional understanding of functional food ingredients, shifting the focus from viable microorganisms to include their inanimate forms and derived cellular components (Collado et al. 2019; Salminen et al. 2021a, 2021b; Vinderola et al. 2022a). After extensive research conducted on postbiotics, there is a shift in the use of health‐promoting metabolites from live organisms (Scott et al. 2022).

The composition of postbiotics is remarkably diverse, encompassing a wide range of bioactive components such as short‐chain fatty acids (SCFAs), microbial cell wall fragments, exopolysaccharides (EPs), cell lysates, teichoic acids, and vitamins (Pimentel et al. 2023; Thorakkattu et al. 2022; Zhong et al. 2024). These components exhibit a variety of biological activities, including immunomodulation, gut barrier enhancement, and anti‐inflammatory effects. SCFAs, including acetate, propionate, and butyrate, are particularly notable for their roles in metabolic regulation, gut epithelial health, and appetite control (Amobonye et al. 2025; Asefa et al. 2025; Wei et al. 2024). Evidence further supports the role of gut‐derived acetate in modulating brain functions and influencing appetite through central metabolic mechanisms (Salminen et al. 2021a). In addition to SCFAs, EPs and microbial cell wall fragments strengthen mucosal barriers and present potential antioxidant and antimicrobial properties, thereby expanding their applications in functional foods with specific health claims (Thorakkattu et al. 2022). However, the varying efficacy of different components underscores the need for deeper research into how these substances work individually and synergistically. Advanced analytical methods, particularly omics‐based approaches, are essential to unravel the complexity of postbiotic compositions and their multifaceted effects on host health (Wei et al. 2024).

Postbiotics offer notable advantages over traditional probiotics and prebiotics, particularly in terms of product stability, safety, and formulation adaptability (Scott et al. 2022; Stelmach et al. 2024). Unlike live probiotics, which are sensitive to processing and environmental conditions in comparison to probiotics, postbiotics are inherently more stable as they are composed of inanimate entities. Moreover, postbiotics do not rely on cold chain supply management, so there is an advantage over probiotics. This stability reduces the risk of batch‐to‐batch variability and mitigates concerns that arise from cell death during storage and transportation (Rad et al. 2020; Wei et al. 2024). Furthermore, the absence of live cells in postbiotics eliminates the risk of adverse interactions, such as systemic infections or microbial translocation, particularly in vulnerable populations like immunocompromised individuals (Wei et al. 2024; T. Wu et al. 2024; Żółkiewicz et al. 2020). Nataraj et al. (2020) stated a few postbiotic pharmacodynamics features like no bacterial translocation, free from antibiotic resistance genes, easy cell lysis to extract and store, and better interaction of bioactive molecules and epithelial cells. Postbiotics can modulate the immune system directly by interacting with host epithelial cells deprived of any need for colonization. The postbiotics do not interact with the food matrix, consenting to add in foods/other formulations through a wide range of pH and temperatures without compromising the bioactive function (Barros et al. 2020). A similar observation was stated by Collado et al. (2019), that the food composition and processing conditions like water activity, temperature, pH, oxygen content, and the nature of packaging material majorly affect probiotics’ stability and viability. These attributes elevate the safety anprofile of postbiotics significantly. Additionally, their stability facilitates their integration into various food and pharmaceutical formulations, including heat‐treated or shelf‐stable products (Prajapati et al. 2023; Rad et al. 2020; Rafique et al. 2023; Wei et al. 2024). This adaptability enhances the potential for wider industrial applications and supports the development of products with precise dosage control.

Thermal processing remains a cornerstone of postbiotic production due to its established effectiveness in microbial inactivation and bioactive compound extraction. However, the conventional methods, like tyndalization/heat‐killing used for postbiotic production, result in the denaturation of bioactive metabolites like enzymes, surface proteins, SCFAs, and loss of structural integrity of EPs that impact the functionality and reduce the strain‐specificity of postbiotics (Riza Fathima et al. 2024; Zhong et al. 2024). de Almada et al. (2016) reported that the heat‐inactivation method results in DNA damage, depletion of nutrients, ribosome aggregation, inactivation of enzymes, and protein coagulation, which affects the overall cellular structures and in turn the biological activity of the postbiotics. Similarly, heat‐killing induced cell coarseness and roughness, impairing postbiotic immunomodulatory effects and decreased release of SCFA (acetic and butyric acid) with heat‐killing temperatures rising from 65 to 95°C, indicating possible degradation (Müldür et al. 2025). These limitations, including the potential for altering sensitive bioactive and affecting sensory qualities, have driven interest in refining this approach through parameter optimization and integration with other more innovative and nonthermal techniques. Therefore, it is essential to explore and compile information on nonthermal methods of postbiotic application as promising alternatives to preserve their functional properties. Some of the nonthermal technologies, like pulsed electric fields (PEFs), ultrasonication, irradiation, cold plasma, pulsed and ultraviolet (UV)‐light technology, and high‐pressure processing (HPP), are being used in the food processing sector as minimally processed methods that preserve the quality and extend stability without any loss in nutritional profile. Nonthermal technologies are chemical‐free and require less energy than thermal methods, contributing to sustainability. These nonthermal technologies easily facilitate the disruption of probiotic cell walls, releasing the intracellular components. Recent advances in both postbiotic research and nonthermal processing technologies reveal a rapidly evolving field with notable opportunities for further innovation. HPP and PEFs have demonstrated efficacy in microbial inactivation and the enhancement of bioactive compound extraction. Ultrasound and cold plasma are garnering increasing attention for their capacity to preserve thermally sensitive postbiotic constituents and improve product attributes. Notwithstanding these developments, several challenges persist: scaling up these methods, refining process parameters, and addressing regulatory as well as economic constraints remain ongoing considerations. The successful integration of nonthermal technologies into industrial food manufacturing requires further evidence and evaluation to confirm their feasibility, cost‐effectiveness, and compliance with relevant safety standards. Despite their potential, these nonthermal methods remain underexplored in the context of postbiotic production, with many critical aspects yet to be fully understood or optimized.

The central research question addressed in this review investigates whether emerging nonthermal technologies have the capacity to revolutionize the production of postbiotics and whether they are suitable for scaling up to industrial applications. By critically analyzing this question, this review also fills a crucial gap in the current literature by synthesizing interdisciplinary insights from microbiology, food technology, and biotechnology to evaluate the efficiency, effectiveness, and scalability of nonthermal postbiotic production techniques. This review is both timely and innovative due to the growing global emphasis on developing sustainable and effective postbiotic production methods that retain maximum bioactivity and functional benefits. It also addresses the scarcity of comprehensive assessments that focus explicitly on how these methods impact postbiotic stability, bioactivity, and safety. An additional focus is placed on evaluating their potential for adoption on an industrial scale and the specific advantages they offer over conventional thermal processing. Key nonthermal technologies discussed include HPP, PEFs, ultrasound, cold plasma, and UV radiation. Their respective principles, mechanisms, and industrial applicability are examined for their influence on the bioactivity, stability, and functional value of postbiotics, with comparative and critical evaluations elucidating their individual strengths, limitations, and possible avenues for optimization. Additionally, the review identifies significant research gaps, such as the lack of standardized protocols for comparing nonthermal approaches, limited data on long‐term functional preservation of postbiotics, and challenges in scaling up processes for industrial application. By critically examining these gaps, the review not only distinguishes itself from prior works but also provides a clear roadmap for future research, development, and practical implementation. This review paper is structured to guide the reader through the foundational aspects of postbiotics and their production, followed by a critical and focused comparison of traditional and nonthermal production methods regarding their efficiency and effectiveness in postbiotic production and will serve as a valuable resource for scientists, and technologists, aiming to harness the full potential of postbiotics through innovative nonthermal production strategies.

## 2 Postbiotics: Components, Health Benefits, and Applications

**FIGURE 1:**
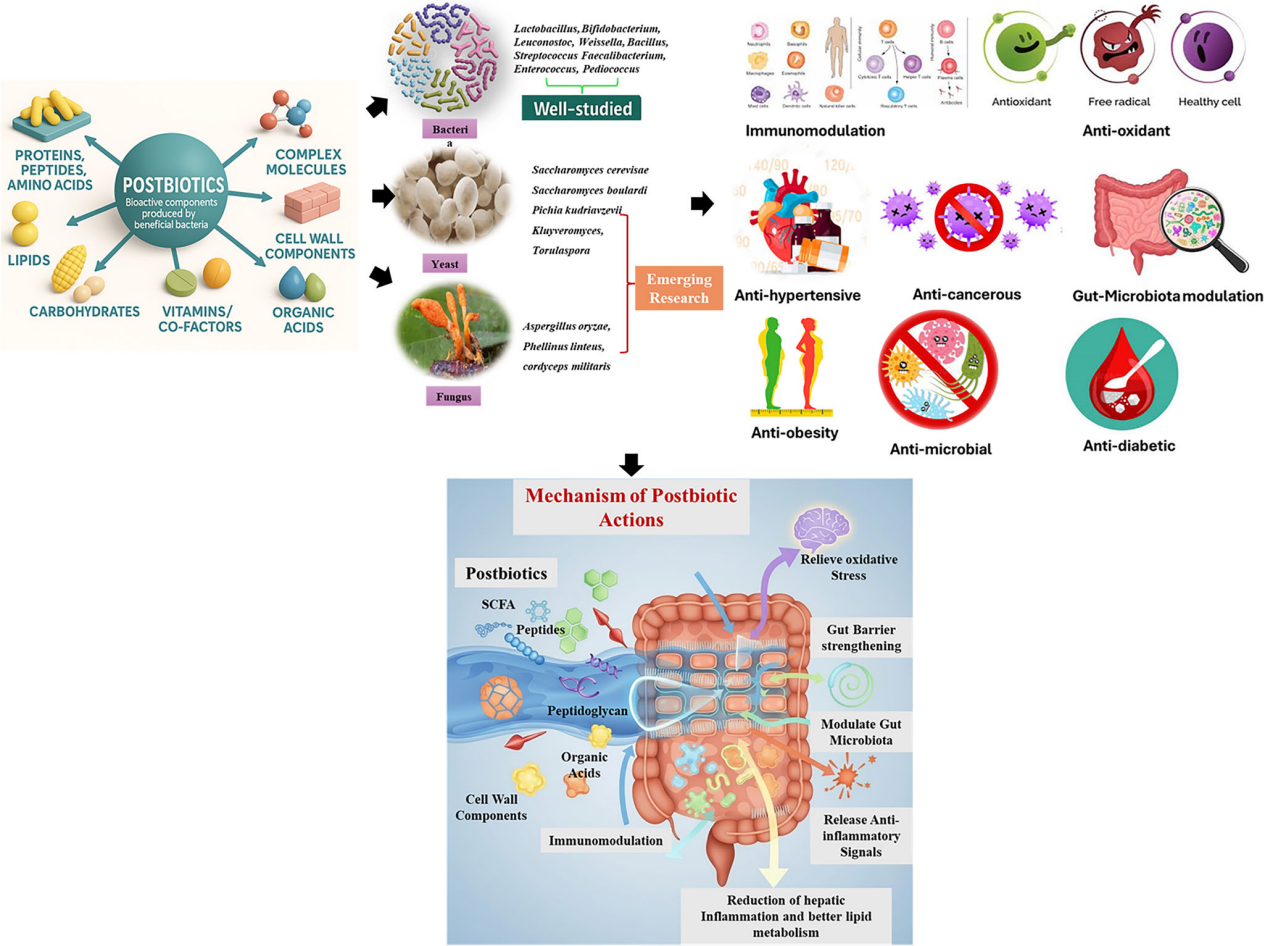
Sources for postbiotics production, their reported health benefits and possible mechanism of actions.

Lactic acid bacteria fermentation produces various health‐promoting cellular components, and metabolites are produced. Postbiotics possess health benefits such as immunomodulatory, anti‐inflammatory, anticancer, antimicrobial, antioxidants, hypocholesterolaemic, anti‐hypersensitive. The postbiotics are categorized into microbial metabolites (lipids, proteins, EPs, organic acids, and enzymes etc.) and components (teichoic acid, peptidoglycan, and cell surface proteins) (J. Aguilar‐Toalá et al. 2018). They can also be classified based on structure and composition (Thorakkattu et al. 2022). These postbiotics interact with the host and provide therapeutic approaches for systematic and local effects (Figure 1). The role of postbiotics as active pharmaceutical ingredients is given below.

### 2.1 Exopolysaccharides

EPs are the microbial metabolites released outside the bacteria cell wall during fermentation, which mainly help in bacterial adhesion. EP is produced extracellularly and attached to microbial cells as a slime layer influencing the host immunity, lipid metabolism, and pathogen colonization (Aggarwal et al. 2022). These are classified as biopolymers with long‐chain branched homo or heteropolysaccharides. The main constituents of EPs are lipopolysaccharides and peptidoglycans with different sugars like glucose, galactose, glucans, arabinose, xylose, mannose, and galactose. EPs exhibit bioactive properties as antioxidants, anti‐cholesteremic, anti‐obesogenic and have immunomodulatory effects (Hijová 2024). An in vivo study showed that the intake of EP produced from *Lactobacillus (Lb.) plantarum L‐14* ameliorated obesity‐associated diseases and was helpful in obesity treatment (Hijová 2024) (Table 1). EPs are widely used in the food, dairy, and pharmaceutical industries as stabilizers and emulsifying agents to modify rheological properties. EPs possess immunomodulatory effects by interacting with macrophages and dendritic cells, modulating the activity of T and NK lymphocytes (Żółkiewicz et al. 2020). EP stimulates lymphocyte proliferation by enhancing the production of IgA in the intestinal mucosa (Y. Wang et al. 2017). Prajapati et al. (2023) reported that β‐glucan can enhance cellular immunity to microbial infection by binding to Dectin‐I receptors of macrophages surface. It has been reported that EP is known to regulate the production of the cytokine response and Th1 pathway inhibition (J. Aguilar‐Toalá et al. 2018). W. Li et al. (2014) reported that uronic acid, a derivative of EP, found during the *Lb. helveticus* fermentation showed similar antioxidant activity to green tea by binding iron. Kefiran is one of the EP produced by *Lactobacillus*, demonstrating anti‐cardiovascular activity and decreased blood pressure during the animal trials (Żółkiewicz et al. 2020). However, EPs production mainly depends on the strain, media composition, pH, temperature, and cell age during fermentation (Nataraj et al. 2020).

**TABLE 1:** Recent scientific data on postbiotic health benefits published during the last 5 years.

**Microorganism**	**Postbiotic molecule**	**Health benefits**	**References**
*Lb. fermentum L‐14*	Exopolysaccharide	Anti‐obesity	J. Lee et al. (2020)
*Lactobacillus* sp. *(La1 and La2)*	Cell‐free supernatant	Antioxidant activity	Y. Kim et al. (2022)
*Lb. paracasei D3‐5 strain*	Lipoteichoic acids	Antiaging	S. Wang et al. (2020)
*Lb. fermentum*	Cell‐free supernatant	Immunomodulation	R. Kumar et al. (2020)
*Lb. animalis*	Bacterial vesicles	Reduction of cell apoptosis	C.‐Y. Chen et al. (2022)
*Lb. plantarum*	Extracellular vesicles	anti‐inflammatory effect	Hao et al. (2021)
*Lb. fermentum*	Cell‐free supernatants	Antiviral effects against herpes	Vilhelmova‐Ilieva et al. (2022)
*Lb. plantarum*	Short‐chain fatty acid and lactic acid	Modulated the intestinal bacteria	Y. Li et al. (2023)
*Lb. rhamnosus GG* *Lb. reuteri*	Cell‐free supernatant	Antimicrobial affect	Banakar et al. (2023)
*Lb. helveticus 611*	Cell‐free supernatants	Antibacterial and antifungal activity	Dobreva et al. (2024)
*Lb. paracasei*	Cell‐free supernatants	Modulating a host immune response	Rossoni et al. (2020)
*Lb. plantarum RM1*	Cell‐free supernatants	Aflatoxin M_1_ decontamination	Mogahed Fahim et al. (2021)
*L. rhamnosus CRL1505*	Peptidoglycan	Immunomodulatory	Salva et al. (2021)
*Lb. acidophilus LA‐5*	Heat killed cells	Antioxidation property and anticancer	Yavaş et al. (2024)
*Lb. curvatus B.67*	Bacteriocin	Antimicrobial activity	Hossain et al. (2021)
*Lb. casei* subsp. *casei PTCC 1608*	Bacteriocin	Antibacterial and anti‐virulence properties	Azami et al. (2022)
*Lb. plantarum*	Cell‐free supernatants	Immunomodulation	Y. Wu et al. (2023)
*Lb. paracasei*	Cell‐free extract	Antioxidant activity	Osman et al. (2021)
*Bifidobacterium (B.) bifidum MG731*	Heat‐killed isolates	Anti‐inflammatory potential	C. H. Kang et al. (2021)
*Schleiferilactobacillus harbinensis LH 991* *Pichia kudriavzevii B‐5P*	Short‐chain fatty acids	Antimicrobial activity	Marlida et al. (2024)
*B. longum CECT 7347*	Short‐chain fatty acids	Anti‐cholesteromic	Naghibi et al. (2024)
*Faecalibacterium prausnitzii*	Short‐chain fatty acids	Gut microbiota modulation	Maiuolo et al. (2024)
*Lb. plantarum*	Cell‐free supernatants	Anti‐inflammatory activity	El Far et al. (2023)
*B. bifidum* *Lb. plantarum DSA 20174* *Lb. acidophilus* *Lb. helveticus CNRZ 32* *Lb. rhamnosus GG*	Vitamins, organic acid, and short‐chain fatty acids	Antimicrobial, antioxidant activities	G. A. Ibrahim et al. (2025)
*Limosilactobacillus reuteri*	Short‐chain fatty acids	Antibacterial properties	Jalali et al. (2024)
*Lb. parabuchneri MF2103 *	Short‐chain fatty acids	Gut microbiota modulation	Fang et al. (2023)
*Lactiplantibacillus plantarum*	Organic acids	Antimicrobial activity and antioxidant	Chang et al. (2021)
*Enterococcus faecium*	Bacteriocin	Antimicrobial activity	Popović et al. (2023)
*Enterococcus faecalis*	Bacteriocin	Antimicrobial activity and anti‐spore germination activity	Luenglusontigit et al. (2023)
*Lb. plantarum*	Cell‐free supernatants	Antibacterial and anti‐biofilm	Nezhadi and Ahmadi (2024))
*Lb. plantarum EIR/IF‐1*	Cell‐free culture media	Antimicrobial activity	Karaca et al. (2023)
*Saccharomyces boulardii*	Freeze‐dried and spray‐dried	Preventing ulcerative colitis	Jin et al. (2025)
*Bacillus subtilis H4 and Bacillus amyloliquefaciens LFB112*	Mulberry leaves fermentation	Anti‐inflammatory and antioxidant	Z. Abbas et al. (2024)
*Lacticaseibacillus rhamnosus (Lc. rhamnosus) GG and Lactiplantibacillus plantarum (L. plantarum) 299v*	Thermally inactivated cultures	Immunomodulatory properties	Mosiej et al. (2025)
*Lb. plantarum PTCC1745*	Thermally inactivated cultures Ultrasonicated cultures	Antibacterial and antifungal	Khakpour et al. (2024)
*Lacticaseibacillus paracasei SNB*	Capsular polysaccharide Surface layer protein	Improved intestinal barrier dysfunction and gut microbiota	Xiao et al. (2024)
*Lacticaseibacillus casei 01*	Ohmic heating inactivated cultures	Hypoglycemic activity via inhibition of α‐glucosidase and α‐amylase	Barros, Grom, et al. (2021)
*Lb. paracasei* Shirota	Cell free supernatant	Biosurfactants/bioemulsifiers, lipase, and bacteriocins	de Medeiros et al. (2024)
*Lb. plantarum* subsp*. plantarum* and *Bifidobacterium animalis* spp. *lactis BB‐12*	Cell free supernatant	Antimicrobial action	Khorshidi et al. (2025)
*Phellinus linteus*	Galacturonic acid‐rich polysaccharide	Immunomodulatory properties	Suh et al. (2023)
*Cordyceps sinensis Cs‐HK1*	EPS	Anti‐inflammatory activities	L. Li (2022)
*Cordyceps militaris*	Polysaccharides	Enhanced aggregation properties and metabolite production by probiotics	J. Y. Kang et al. (2024)

### 2.2 Short‐Chain Fatty Acids

SCFAs are the most important and widely studied of several metabolites with different therapeutic activities. SCFAs are volatile organic metabolites primarily produced by colon bacterial fermentation of plant polysaccharides (prebiotics). SCFAs (acetate, propionate, and butyrates) are produced by the gut microbiota through dietary fiber fermentation (Omak and Yilmaz‐Ersan 2022; Ragavan and Hemalatha 2024). Prebiotic fermentation products of fructooligosaccharides (FOS) and inulin are rich in SFCs like acetate, propionate, and butyrates (Prajapati et al. 2023). A high level of SFC (23 mg/g) was observed in the probiotic extract of *Lb. rhamnosus* (Jalali et al. 2024). Mousavi Ghahfarrokhi et al. (2024) stated that butyrate is an energy source for erythrocytes that are responsible for restoring intestinal epithelium and modulating gene expression. SFC modulates G‐coupled protein receptors and inhibits histone deacetylases, regulating the anti‐inflammatory effect through signaling pathways (Table 1) (Z. Zhou and Chen 2025). These are necessary for maintaining immunological and gastrointestinal homeostasis. The gut microbiota produces the SCFA, which regulates glucose metabolism and reduces blood glucose levels, which benefits diabetic patients (X. Chen et al. 2022). The SFC's anti‐inflammatory activities are due to the monocytes and blood mononuclear cells secreting prostaglandins, cytokines, and chemokines (Hijová 2024). Mosca et al. (2022) reported that postbiotics, particularly the SCFAs, mitigate cardiovascular disease by decreasing oxidative stress, cholesterol levels, and inflammatory processes. Ragavan and Hemalatha (2024) proposed the immunomodulation mechanism of CFAs by regulating the IL‐10 and IL‐18 functions, activating E1 and E2 prostaglandin production that enhances the mucin production helpful to protect from infections. The clinical investigation by van Beek et al. (2024) observed that gut microbe‐derived acetate SCFAs altered whole‐body substrate metabolism with increased fasting fat oxidation, reducing the cardiometabolic risk factors. The case study reported by A. Kumar et al. (2024), results suggested that sodium butyrate as a postbiotic decreased the severity of abdominal pain and other symptoms related to irritable bowel syndrome (IBS), improving patient quality of life. A similar statement regarding IBS was reported by Ragavan and Hemalatha (2024), the SCFAs act as a therapeutic option for IBS patients by regulating the function of gut microbiota.

### 2.3 Cell‐Free Supernatants (CFS)

The CFS obtained from the probiotic strains of *Lactobacillus* are rich in antioxidants and anti‐inflammatory, anti‐microbial, and anti‐cancer components. These bioactive metabolites are produced during fermentation and are collected by centrifugation. Hijová (2024) reported that biomolecules and metabolites that are obtained by centrifugation of cell cultures possess human health benefits (Table 1). Apart from these metabolites, hydrogen peroxide, proteins, diacetyl, and lactic acid are observed in measurable quantity (George‐Okafor et al. 2020). The biofilms prepared with the incorporated *Lb. gasseri* postbiotics showed significant amounts of polyphenol with strong antioxidant and antibacterial properties (Ceylan 2024). CFS of *Lb. fermentum, Lb. paracasei*, and *Lb. brevis* contained higher levels of polyphenols, and flavonoids exhibited anti‐oxidant, anti‐biofilm activity, and anti‐inflammatory properties (Sornsenee et al. 2024). Similarly, the flavonoid content (1971.79 ± 20 mg Qu/g extract) was observed in the probiotic extract of *Lb. rhamnosus* (Jalali et al. 2024). Chang et al. (2021) reported the production of lactic acid and acetic acid during the production of *Lb. plantarum* postbiotics. In a similar study on *Lb. plantarum* postbiotic, Rocchetti et al. (2024) reported the CFS‐modulated cytokine pattern and promoted the production of IL‐10. The cell‐free extract of *Lb. fermentum* showed anticancer properties on colorectal cancer cells (J. Lee et al. 2020). *Lb. casei CRL*431 postbiotic supernatant decreased the metastasis properties of colon cancer with significant antigenotoxic (10%–50%) and cytotoxic potential (70%–80%) (Abbasi et al. 2022). Shin et al. (2024) observed the anti‐obesity effect with *Bacillus velezensis KMU01* cell‐free extract. Cell‐free *Lactobacillus* supernatants reduced fungal viability and metabolic activity and improved epithelial resistance to fungus (Spaggiari et al. 2022). CFS's anti‐oxidant capacity is greater than the intact whole cell culture (Bourebaba et al. 2022).

### 2.4 Peptides

Most of the intestinal *Lactobacillus* bacteria produce peptides that possess anti‐microbial properties that are effective in fighting against bacterial and viral infections. These anti‐microbial peptides inhibit bacterial action by creating pores in the cell walls. Waghu and Idicula‐Thomas (2020) proposed that the anti‐microbial peptide inhibits macromolecular synthesis and degrades the microbial membrane. The anti‐microbial peptide mechanism proposed by Prajapati et al. (2023) includes acidification of cell membrane, cell membrane poration, production of certain enzymes that are fatal to cells, and damage to internal cell organelles. Bacteriocins produced by LAB are cationic peptides that mainly affect the cytoplasmic membrane through pores, resulting in cell leakage (Bourebaba et al. 2022). Wegh et al. (2019) reported that bacteriocins are ribosomal peptides with bactericidal and bacteriostatic effects. Bacteriocins are a class of peptides that are associated with several food safety applications (Cheruvari and Kammara 2024). Hols et al. (2019) stated that bacteriocins benefit through the six fundamentals, which include stability, spectrum, safety, variety, bioengineering, and production. Of the several bacteriocins, nisin and pediocin are the most widely used postbiotics in food applications. O'Connor et al. (2020) reported that bacteriocins have been employed in fermented food for ages. The postbiotic surface proteins showed several anti‐inflammatory properties, absorbed harmful heavy metals, and strengthened the epithelial barrier function (Prajapati et al. 2023). Postbiotic peptides are also used to prepare packaging materials with antimicrobial properties (Aggarwal et al. 2022). Nataraj et al. (2020) reported that the surface protein extract of *Enterococcus faecium* inhibited the apoptosis of Caco‐2 induced by *Listeria monocytogenes*.

### 2.5 Vitamins and Enzymes

Vitamins are important postbiotics produced by the gut microbiota. Humans do not biosynthesize vitamins; they are acquired through food, and some vitamins like B12 are produced by gut microbiota. Some LABs can synthesize vitamin B2 and folic acid (G. A. Ibrahim et al. 2025). Prajapati et al. (2023) reported that *Propionibacterium freudenreichii* 2067 can synthesize B12. On the industrial scale, the enzymes like proteases derived from the *Bacillus subtilis* are of great interest (Rafique et al. 2023). Many of the vitamins act as coenzymes in several metabolic pathways. The other class of postbiotic metabolites is enzymes. The enzymes related to the defense mechanism are important and play a role in combating free radicals. Enzymes derived from bacteria and fungi are industrially important. Probiotic enzymes like superoxide dismutase, NADH‐oxidase, metalloenzymes, glutathione peroxidase, and catalases are known to fight against reactive oxygen species (Prajapati et al. 2023). Catalase, as a postbiotic enzyme from *Lb. lactis*, inhibited colon cancer in mice (Thorakkattu et al. 2022).

## 3 Factors Affecting Postbiotic Production During Fermentation

### 3.1 Fermentation Substrate

Fermentation techniques and substrates enable the targeted production of postbiotics by leveraging specific microbial strains grown under conditions optimized for this purpose (Z. Abbas et al. 2024; Amiri et al. 2021). The choice of fermentation substrate substantially influences not only the yield but also the functional properties of the resulting postbiotics (Z. Abbas et al. 2024). Additionally, the type of substrate used during fermentation directly affects the spectrum and concentration of metabolites produced, demonstrating a significant influence on the bioactive properties of the resulting postbiotics.

Leveraging diverse fermentation substrates, such as MRS broth, milk proteins, cheese whey, or renewable bio resources alongside innovative microbial strains, can enhance both the spectrum and potency of bioactive metabolites in postbiotics (Danova et al. 2023; Khakpour et al. 2024; Ma et al. 2023; Ooi et al. 2021). These fermentation substrates also differ in their ability to support microbial growth and facilitate the production of different bioactive compounds (Khakpour et al. 2024; Sadighbathi et al. 2023; T. Wu et al. 2024). Observed evidence highlights that MRS broth generally supports greater bacterial proliferation compared to milk and demonstrates comparable performance to whey (Khakpour et al. 2024). However, MRS broth produced postbiotics showed superior anti‐microbial properties exhibit larger inhibition zones against pathogenic bacteria than those derived from milk or whey, indicating substrate‐dependent variations in anti‐Salminen et al.microbial efficacy. MRS‐derived postbiotics also showed more favorable nanoparticle characteristics compared to milk or whey, which are critical to the bioavailability and functional application of postbiotics in food and pharmaceutical systems (Khakpour et al. 2024). In another study, MRS medium supplemented with ribose improved the antimicrobial activity of postbiotic obtained from *Pediococcus acidilactici* CECT 9879 and *Weissella cibaria* CECT 30731 in comparison to glucose‐based MRS medium (Garrote Achou et al. 2025). These findings emphasize the critical role of substrate selection in shaping the functional attributes of postbiotics, particularly in industrial production settings where reproducibility and efficiency are key considerations. Variability in substrate performance suggests the need for a systematic approach to substrate evaluation, ensuring that the chosen medium aligns with production goals and intended applications. Therefore, further exploration of alternative substrates and their impact on metabolite profiles would contribute to a deeper understanding of the functional diversity of postbiotics (H.‐J. Kim et al. 2024).

Furthermore, the utilization of fermentation substrates for postbiotic production also presents a unique opportunity for industrial upcycling and food waste valorization (Chávez‐Alzaga et al. 2024; Vera‐Santander et al. 2024). By converting abundant industrial byproducts into high‐value functional ingredients, it aligns with broader sustainability goals and contributes to waste reduction. This is indeed true particularly in the case of whey, a byproduct of dairy production, as whey serves as an effective fermentation substrate, producing postbiotics with functional properties comparable to or exceeding those derived from more conventional media (Chávez‐Alzaga et al. 2024). Similarly, Danova et al. (2023) demonstrated postbiotic with significant antimicrobial function from media supplemented with dried distillers’ grains, wastewaters from rose oil distillation industry as a source of carbon. Furthermore, whey–grape juice was used for production of antihyperglycemic paraprobiotics from *Lacticaseibacillus (Lc.) casei* 01 (Barros, Grom, et al. 2021). In another study, researchers utilized green tea residue waste for production of postbiotics through probiotic anaerobic digestion (Lee et al. 2023).

However, the scalability and consistency of using dairy, agro, and other food waste as a substrate for postbiotic production requires further validation. The integration of food waste into industrial postbiotic production systems could also prompt regulatory challenges due to their status as a byproduct, necessitating clearer guidelines for their utilization in food‐grade applications (Bhatia et al. 2024).

### 3.2 Microbial Strains and Consortia

In addition to fermentation substrates, optimizing microbial strains and their consortia within selected fermentation substrates provides another avenue for enhancing the targeted production of postbiotics using precision fermentation (Perez et al. 2020; Prajapati et al. 2023). Till date various microorganisms, such as bacteria (*Lactobacillus, Bifidobacterium, Leuconostoc, Weissella, Bacillus, Streptococcus Faecalibacterium, Enterococcus*, and *Pediococcus)*, fungus (*Aspergillus oryzae, Phellinus linteus, and cordyceps militaris*), *and yeasts (Pichia, Kluyveromyces, Torulaspora*, and *Saccharomyces*), are the most widely used for production of postbiotics (Figure 1) (Franco 2024; J. Y. Kang et al. 2024; J. P. Mehta et al. 2025; Sadeghi et al. 2022; Seidler et al. 2024; Suh et al. 2023). Recent investigations suggest that distinct microbial dynamics can augment the overall bioactivity of postbiotics, thereby enabling the development of formulations with specific health benefits. In this direction strategic microbial pairing, as exemplified by the coculture of *Bacillus subtilis* H4 and *Bacillus amyloliquefaciens* LFB112, which significantly increased the antioxidant capacity and antibacterial activity of mulberry leaves derived postbiotics, underscore the importance of investigating various microbial consortium (Z. Abbas et al. 2024). In a similar study, the anti‐inflammatory activities of postbiotics produced from edible mushroom *Cordyceps sinensis Cs‐HK1* were further improved by *Bifidobacterium* fermentation (L. Li 2022). However, achieving consistency in these outcomes depends on rigorous control over fermentation variables such as inoculum ratio, environmental conditions, and nutrient availability. Further research is, therefore, warranted to explore whether these findings are generalizable across other microbial strains and fermentation systems (Hernández‐Velázquez et al. 2024; Perez et al. 2020).

### 3.3 Fermentation Method

The type of fermentation such as submerged (batch, fed batch) or solid‐state fermentation is another factor to consider for optimized production of postbiotics. The precise control over environmental conditions has been shown to maximize metabolite production and their biological function. In one such study mulberry‐derived postbiotics produced under optimized submerged fermentation conditions demonstrated enhanced antioxidant activity and reduced inflammatory markers in vitro (Z. Abbas et al. 2024). However, in another study on solid‐state fermentation by *B. amyloliquefaciens* J and *Lc. plantarum* SN4 showed enhanced antibacterial, antioxidant, and anti‐inflammatory activities (Tong et al. 2023). However, a comparative analysis between the superiority of both fermentation techniques remains underexplored and the increased complexity of both methods necessitates advanced monitoring and feedback systems to ensure consistent bioactivity across production cycles.

Further, advancements in sensing technologies can enable real‐time monitoring during fermentation and can provide critical means of ensuring product quality and consistency. These advancements in sensing technologies can help in adjustment of fermentation parameters to maintain optimal conditions for microbial growth and metabolite production mitigating batch‐to‐batch variability (Z. Abbas et al. 2024; Abbasi et al. 2022). However, the adoption of real‐time monitoring technologies is often limited by high costs and the need for specialized expertise, posing barriers to their widespread use in industrial settings. Further advancements in accessible and cost‐effective monitoring tools could enhance the reliability of fermentation‐based postbiotic production (Siddiqui et al. 2023).

ISAPP consensus reinforces that fermentation processes and subsequent inactivation methods must adhere to specific standards to align with the definition of postbiotics as preparations of inanimate microorganisms and/or their components that confer health benefits (Salminen et al. 2021a; Vinderola et al. 2022a). The consensus also underscores the importance of rigorous process validation to ensure product safety, bioactivity, and compliance with regulatory guidelines. The ISAPP consensus further highlights that regulatory clarity is essential for positioning postbiotics correctly within the broader landscape of functional food and nutraceuticals (Salminen et al. 2021a, 2021b; Vinderola et al. 2022a). Establishing universally accepted definitions and standards will facilitate industrial growth while ensuring consumer safety and trust. These requirements stress the wider implications of fermentation optimization, linking scientific advancements with regulatory frameworks to ensure consistency and reproducibility across the industry.

The adoption of clean label approaches in postbiotic production, which emphasizes the use of natural and minimally processed ingredients, aligns fermentation‐derived postbiotics particularly well‐suited to this approach, offering benefits such as natural microbial growth control and preservation of bioactive compounds. However, clean label production methods face challenges, including higher costs and technical limitations associated with scaling up fermentation‐based systems (Constantin et al. 2024; Fernandes et al. 2025). Additionally, regulatory ambiguities surrounding postbiotic definitions and health claims complicate their industrial adoption. Addressing these challenges requires a collaborative effort involving regulatory alignment and technological innovation to ensure that clean‐label postbiotic products are both successful and economically viable.

In brief, fermentation techniques represent a cornerstone of postbiotic production due to their capacity for targeted metabolite synthesis and substrate versatility. However, the variability introduced by substrate and strain‐specific factors necessitates further research and optimization to achieve consistent outcomes. Moreover, although fermentation offers vast potential for refining postbiotic functionalities, its adoption must be supported by advancements in monitoring systems, substrate utilization, and regulatory frameworks to fully harness its industrial applicability.

## 4 Production Methods for Postbiotics

Effective production of postbiotics relies on a variety of innovative methods designed to optimize bioactive compound yield, safety, and functionality. As the field advances, balancing efficacy, safety, and industrial feasibility remains central to transforming scientific insights into practical applications. Therefore, this section investigates techniques that ensure the retention of key health‐promoting properties while enabling scalable, sustainable manufacturing processes by exploring both conventional and emerging technologies.

### 4.1 Conventional Thermal Processing Methods

Conventional methods are primally used in the production of postbiotics that ensure microbial inactivation and bioactive compound extraction. These are well versed in developing stable and safe postbiotic products, serving as a bridge between innovative technologies and practical industrial applications. Their optimization is essential for advancing the field within the broader context of postbiotic research and industry implementations (Pimentel et al. 2023; Zhong et al. 2024).

Thermal processing has long been regarded as one of the most prevalent and cost‐effective methods to produce postbiotics due to its efficacy in microbial inactivation and its ability to enhance the release of bioactive compounds (Abitha Eswari et al. 2024; Sun et al. 2023). Heat treatment, such as pasteurization, autoclaving, and ohmic heating, when applied to probiotic microbial cultures, has been observed to facilitate the extraction of anti‐microbial, immunomodulatory, and anti‐oxidant metabolites effectively (Khakpour et al. 2024; Mosiej et al. 2025; Zhong et al. 2024). The degree of microbial inactivation is significant in food and pharmaceutical applications, where microbial contamination poses a substantial risk (Hassoun et al. 2020; Salminen et al. 2021a). Moreover, some studies have also demonstrated that thermal processing can yield higher levels of bioactive antimicrobial compounds compared to other techniques like centrifugation or sonication, underlining its capacity to extract functional molecules critical for postbiotics (Khakpour et al. 2024).

The conditions of thermal processing, including temperature and duration, and strain type play a role in both the postbiotics yield and the physicochemical characteristics (Abitha Eswari et al. 2024; Pimentel et al. 2023; Zhong et al. 2024). For instance, a comparative analysis between *Lb. acidophilus*, *Lc. casei*, and *Bifidobacterium (B.) animalis* revealed significant difference in their inactivation profile with *Lc. casei* and *B. animalis* showing more thermal resistance than *Lb. acidophilus* at 95°C (Barros, Pires, et al. 2021). Similarly, different inactivation rates are observed at different temperatures for *Lc. plantarum* MIUG BL21 between 60 and 90°C (Stănciuc et al. 2024). Therefore, strain specificity, intensity, and duration of heat treatment must be carefully balanced to achieve microbial inactivation while minimizing undesirable changes in food properties (de Almada et al. 2016). Moreover, the thermal processing can also influence the textural and particle size profile of obtained probiotics that in turn can impact their inclusion in different food applications. For example, applying thermal treatments in the range of 70–100°C has been shown to influence the nanoparticle size distribution of postbiotics (H.‐L. Huang et al. 2024). Higher temperatures can produce smaller, more uniform particles, which may facilitate better dispersion and absorption when incorporated into food matrices (H.‐L. Huang et al. 2024; Khakpour et al. 2024). By varying the temperature and treatment times can result in postbiotics with distinct functional profiles (Ma et al. 2023). High temperatures adversely affect the sensory and nutritional qualities of the final product. High‐temperature treatments can lead to losses in aroma, flavor, color, and essential nutrients, thereby limiting the acceptability of thermally processed postbiotics among consumers (Hassoun et al. 2020; Pham et al. 2024). The application of heat also poses a risk of altering the structural integrity of heat‐sensitive bioactive substances such as peptides and SCFAs (Miao et al. 2024). These structural alterations could diminish their biological activity or modify their health‐promoting properties (Ma et al. 2023; Sun et al. 2023).

The regulatory framework for postbiotics, which emphasizes the inclusion of inanimate microbial cells or their components, aligns well with the objectives of controlled thermal inactivation. Heat treatment is particularly well‐suited for meeting these regulatory requirements by effectively neutralizing microbial cells without compromising the fundamental definition of postbiotics (Salminen et al. 2021a). However, the lack of consensus on the specific conditions needed to achieve both complete microbial inactivation and functional efficacy complicates the standardization of thermal processes. Therefore, addressing these regulatory and methodological uncertainties remains essential for advancing the safe and effective industrial production of thermally processed postbiotics (Homayouni‐Rad et al. 2025).

Overall, although thermal processing remains a cornerstone of postbiotic production due to its established effectiveness in microbial inactivation and bioactive compound extraction. However, its limitations, including the potential for altering sensitive bioactives and affecting sensory qualities, have driven interest in refining this approach through parameter optimization and integration with other techniques. Continued research into the interplay between heat treatment conditions and microbial strains is vital for advancing postbiotic production beyond conventional boundaries. The ongoing evolution of thermal processing techniques underscores its enduring relevance in the field while highlighting the need for innovation to address its current challenges. These include novel approaches such as microwave heating, radio frequency heating, and infrared heating, which offer potential advantages in terms of energy efficiency and reduced processing times compared to conventional thermal methods (Barros, Pires, et al. 2021).

### 4.2 Nonthermal Technologies

Nonthermal technologies offer innovative approaches for producing and enhancing postbiotics production while preserving their bioactive compounds and ensuring microbial safety. These advanced methods ranging from physical to alternative processing techniques play a crucial role in optimizing production processes, improving product stability, and aligning with sustainability and regulatory standards (Jadhav et al. 2021). Further, advancements in nonthermal technologies are revolutionizing the production and application of postbiotics, enabling safer, more effective, and sustainable solutions for health promotion (Riza Fathima et al. 2024). These emerging methods ranging from high pressure and PEFs to ultrasound, irradiation, and cold plasma offer innovative pathways to preserve bioactive compounds while enhancing microbial safety. Within the broader context of postbiotic research, exploring these cutting‐edge applications highlights their potential to shape the future landscape of functional foods and nutraceuticals (Aggarwal et al. 2022; J. Aguilar‐Toalá et al. 2018; Bourebaba et al. 2022; Vinderola et al. 2022a; Wei et al. 2024; Żółkiewicz et al. 2020).

#### 4.2.1 Physical Methods

**FIGURE 2:**
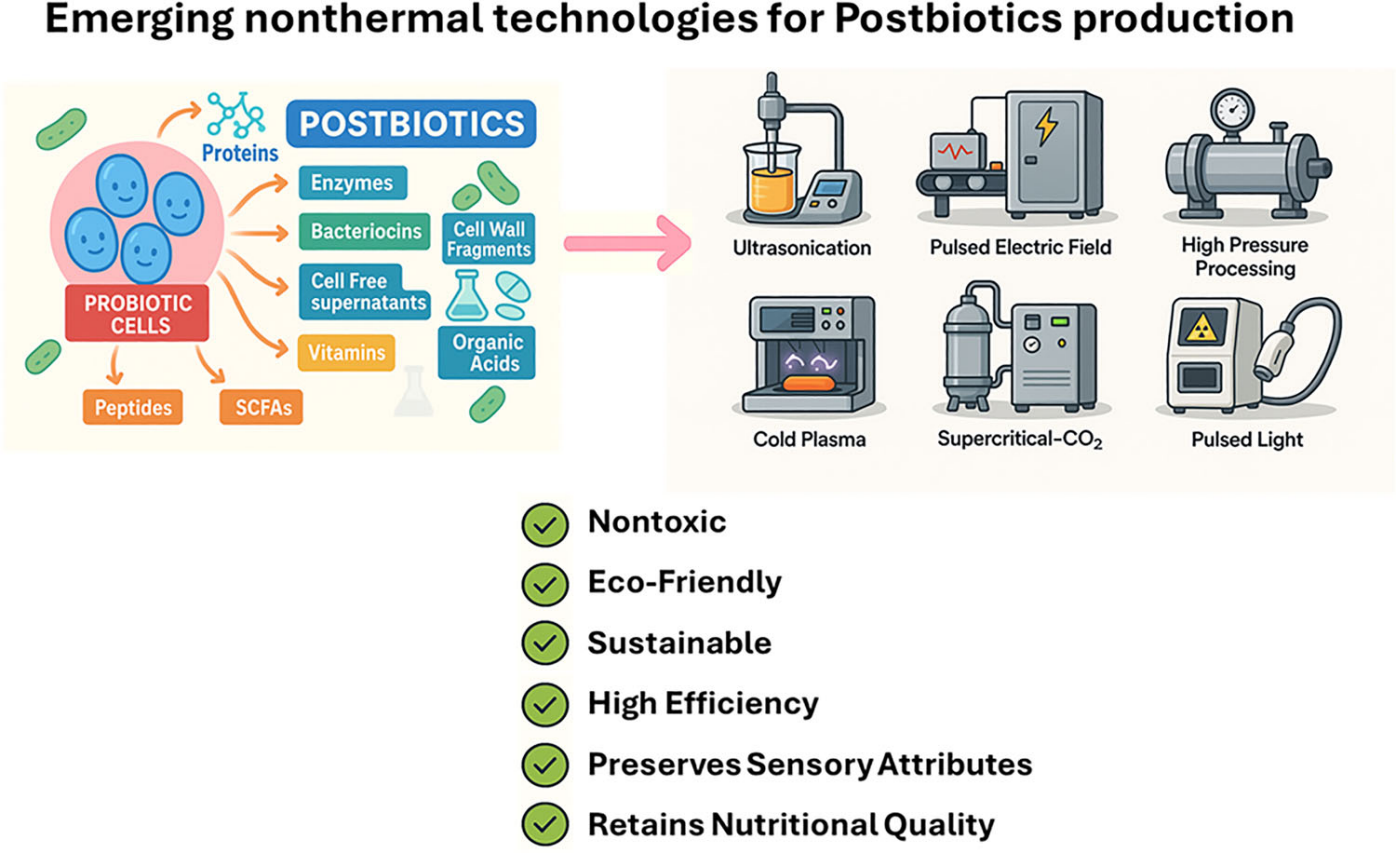
Emerging nonthermal technologies with potential use in postbiotic production.

Advocates of nonthermal physical methods argue that they outperform conventional thermal technologies by preserving postbiotics’ functional and nutritional integrity. Nonthermal physical methods show significant industrial potential in functional food production. Unlike heat‐based treatments, nonthermal techniques like ultrasonication, HPP, and PEFs inactivate microorganisms without thermal degradation of bioactive compounds combining safety, sustainability, and economic advantages (Pimentel et al. 2023; Zhong et al. 2024). This ensures thermally sensitive molecules, such as SCFAs and bacteriocins, retain their properties such as anti‐oxidant capacity and anti‐microbial efficacy (Ashrafudoulla et al. 2023; Hassoun et al. 2020). These technologies also enhance key health‐promoting compounds like SCFAs, bacteriocins, and EPs, important for metabolic health and immune modulation (Asefa et al. 2025). Given growing consumer demand for bioactive‐rich functional foods, nonthermal technologies’ ability to ensure safety while preserving bioactivity makes them preferred over traditional methods (Melios et al. 2025). Further, products derived from nonthermal methods align with the clean label movement, which consumers associate with greater health benefits (Melios et al. 2025; A. Silva et al. 2024; F. V. M. Silva and van Wyk 2021). Their reduced thermal energy use provides economic and environmental benefits (Hassoun et al. 2020). Postbiotics produced through nonthermal processes maintain higher bioavailability, augmenting their host system interaction (Aggarwal et al. 2022; Almada, Almada‐Érix, Bonatto, et al. 2021; Almada, Almada‐Érix, Roquetto, et al., 2021). This retention benefits chronic conditions management and systemic metabolism (Figure 2), whereas preserving postbiotics’ structural features enhances their efficacy in addressing inflammatory and metabolic disorders. These advantages position nonthermal techniques as a bridge between functional foods and biotherapeutics, fostering innovation in personalized nutrition and clinical applications (Asefa et al. 2025; Homayouni‐Rad et al. 2025; Melios et al. 2025).

In summary, nonthermal physical methods offer promising advancements in postbiotic production by enhancing bioactive compound extraction, preserving functional integrity, and supporting sustainability objectives. However, further innovations in scaling, cost reduction, and regulatory alignment are essential to optimize these technologies for widespread industrial application. These physical nonthermal methods are now discussed in more detail below.

##### 4.2.1.1 Ultrasound Technology

Ultrasonication has emerged as one of the most innovative and popular methods among nonthermal physical methods for enhancing postbiotics production. Ultrasonication has been applied before and after fermentation to promote microbial cell disruption and metabolite extraction (Riza Fathima et al. 2024). This process relies on the principles of acoustic cavitation, where high‐frequency sound waves generate microbubbles that subsequently collapse, creating intense localized shear forces capable of disrupting microbial cell walls (Mudgil et al. 2022). The mechanical effects not only facilitate the efficient release of postbiotic constituents, such as biosurfactants, bacteriocins, and EPs, but also enable the processing of complex substrates for microbial utilization, improving the postbiotic production (Behzadnia et al. 2020) (Table 2).

**TABLE 2:** Nonthermal technologies applied for postbiotic production.

**Fermentation substrate**	**Cultures**	**Method**	**Outcome**	**References**
**Ultrasonication and combinations**				
**MRS**	*Lactobacillus* sp. (La1 and La2)	Ultrasonication (20 min, 70% amplitude, and 50 W)	Anti‐proliferation effects against HT‐29 (61% and 51% inhibition)	Y. Kim et al. (2022)
	*Lactobacillus paracasei*	Lysozyme (20 min) followed by ultrasonic disruption (5 min)	Management of metabolic syndrome **@100 mg kg^−1^** (**↓**Total lipids—29%; serum triglyceride—32%; cholesterol—40% **↑**HDL—28%) **@200 mg kg^−1^** (**↓**Total lipids—34%; serum triglyceride—45%; cholesterol—39% **↑**HDL—30%)	Osman et al. (2021)
	*Lb. plantarum (LP)* *Lb. gasseri (LG)*	Sonicated (50 and 100 W for 5 and 10 min)	Strain dependent inactivation for paraprobiotics production	Gholian et al. (2024)
	*Lb. plantarum ZDY2013*	Ultrasound and microwave based synthesis of zinc oxide nanoparticles from fermentation liquid (LFL)	Improved antibacterial action against methicillin‐resistant *Staphylococcus aureus* (MRSA) in comparison to commercial ZnO (LFL‐ZnO 100 µg/mL vs. ZnO 400 µg/mL)	W. Li et al. (2023)
	*Lactiplantibacillus plantarum UCLM56*	Sonication (5 min)	Increased production of GABA (360%), propionic acid (260%), and improved antioxidant activity (200%)	Ramos et al. (2025)
	*Lc. plantarum MIUG BL21*	Ultrasound treatment for 10 min at 100% amplitude	Cytocompatibility and antiproliferative effect (132.04% cell viability in comparison to 92.39% of heat treated postbiotic against HT‐29 cell line)	Stănciuc et al. (2024)
	*Lc. plantarum* *(LS5 and LU5)*	Ultrasound (US) pretreatment (100 W, 30 kHz; 0%, 25%, 50%, and 75% amplitudes, 15 min)	Increased EPS yield (∼40 mg/L at 50% in comparison to ∼27 mg/L at 0% amplitude for strain LS5)	Hashemi et al. (2022)
	*Lb. bulgaricus*	Ultrasound	Improved sausage quality and antimicrobial effects	Sheikhi et al. (2025)
	*Lb. casei CRL 431* *Bacillus coagulans GBI‐30*	Ultrasonication two 30‐min pulses (42 kHz)	ACE inhibitory (>90%), and immuno‐modulatory, chelating (>79%), and antioxidant (ca. 22–57 cellular antioxidant activity units)	J. E. Aguilar‐Toalá et al. (2020)
	*Lb. acidophilus 5* *Lacticaseibacillus casei* subsp*. Paracasei 1* *Bifidobacterium animalis* subsp*. lactis 12*	– Low‐frequency ultrasound treatment—20 kHz 10–120 min @ 792 W (W) per cm^2^ – Irradiation treatment (0.5–7 kGy) – Supercritical CO_2_	Strain dependent metabolism activation (*L. casei* showed highest enzymatic activity (86.2%) and lowest membrane damage (4.6%))	Almada, Almada‐Érix, Bonatto, et al. (2021)
	*Lc. casei* subsp. *paracasei 1* *Bifidobacterium animalis* subsp. *lactis* *Bifidobacterium lactis*	Heat, ultrasound, high pH, low pH, irradiation and supercritical carbon dioxide (CO_2_)	Irradiation and SC‐CO_2_ lowered serum cholesterol; SC‐CO_2_ raised albumin and creatinine, but reduced HDL	Almada, Almada‐Érix, Roquetto, et al. (2021)
	*Lc. plantarum*	10 different combinations of power (20% and 40%) and duration (2, 4, 6, 8, and 10 min)	Strain dependent enhancement in functional properties. Biofilm stability; reduced acidification, no technological drawbacks	Bevilacqua et al. (2024)
	*Lc. casei 01*	20 kHz, 40 min	Improve biochemical and cardiovascular health; increased beneficial microbiota	Brandão et al. (2021)
	*Lc. casei ATCC 393*	57 W, duty cycle 50%, 6 or 8 min	Enhanced hydrophobicity (11.68% and 15.01% after 6 and 8 min, respectively), membrane permeability, and fermentative metabolism	Giordano and Mauriello (2023)
	*Lb. plantarum H6 (L.p H6)*	Ultrasonic cell pulverizer for 5 s and 60 min at 9 s intervals	Improved hypercholesterolemia via gut microbiota and lipid metabolism regulation	Y. Li et al. (2022)
ND	*Saccharomyces boulardii*	Ultrasonication (0, 50, 100, 150, 200, and 250/W)	Enhanced biological functions on early‐weaned lambs Higher level of SIgA (14.9 mg/g) and IL‐10 (3.36 mg/g) in 0.5% postbiotic supplemented feed vs. control	M. Liu et al. (2021)
Skim milk	*Lb. acidophilus (LA‐5, CHR HANSEN) and Lb. helveticus (LH‐B02, CHR HANSEN)*	sonication (30% amplitude‐3 min) 20 kHz	Enhanced bioaccessibility index for DPPH (97.67%), FRAP (88.5%) and ORAC (148.61%) with LA‐5 postbiotic	Bolivar‐Jacobo et al. (2025)
Kefir	*Lactococcus lactis* subsp. *cremoris, L. lactis* subsp. *lactis biovar diacetylactis, L. lactis* subsp. *lactis, Leuconostoc*, and *Streptococcus thermophilus*	High‐intensity ultrasound (HIU), ultra‐thermosonication (UTS)	HIU: control‐like bioactivity UTS: stronger microbial kill	Chávez‐Alzaga et al. (2024)
Cheese whey	*Lb. acidophilus LA‐5*	Chia seed mucilage (CSM) films with LA5 postbiotics ultrasonication 25°C for 30 min at 40 kHz	100% antimicrobial effects against *Escherichia coli* O157:H7 at CSM‐LA5 postbiotic (200 mg/mL)	Mardani et al. (2025)
Skim milk, soy milk, and almond milk	*Lc. plantarum J26*	Pasteurization combined with 400 W ultrasound treatment for 30 min	Reduced triglyceride accumulation (0.99 mg per 10^4^ CFU), gut microbiome modulation, and restored obesity‐induced anomalies Elevated acetate (14.95%), propionate (23.89%), and butyrate 8 (0.31%) levels	Miao et al. (2024)
Whey permeates and sugar cane molasses	*Lb. plantarum ATCC 8014*	Ultrasonication at 28 kHz frequency (power of 100 W)	Biosurfactants with antiviral activity Reduced surface tension (39.95 mN/m) in US samples	Behzadnia et al. (2022)
**Pulsed electric field**				
MRS	*Lacticaseibacillus rhamnosus ATCC 7469* *Lacticaseibacillus paracasei NRRL B‐4564*	Batch PEF treatment: pulse amplitudes—300–2500 V electric field strengths—0.3–25 kV/cm	Increased LA (10%) production and protein release (2.1 µg/mL; *L. rhamnosus* after 8 pulses, 1000 µS and µs 5 kV/cm)	Djukić‐Vuković et al. (2021)
MRS	*Lb. plantarum WCFS1*	Electric field strength of 7.5 kV/cm	Increased intracellular trehalose/lactose content	E. M. J. Vaessen et al. (2019) and E. M. J. Vaessen et al. (2018)
PBS and skim milk	*Lb. delbrueckii* ssp. *bulgaricus* LB‐12 and *Lb. acidophilus* LA‐K	Electric field strength of 1 kV/cm, pulse width of 3 µs, pulse period of 0.5 s., flow rate of 60 mL/min	Increased protease activity	Najim and Aryana (2013)
MRS	*Lb. rhamnosus B 442*	Electric field strength‐3.0 kV/cm, time‐10 min frequency 1 Hz	Increased calcium (7.30 mg/g d.m. at 3 kV/cm), magnesium (2.5 mg/g d.m. at 2 kV/cm), and zinc bioaccumulation (2.85 mg Zn/g d.m.; 164% higher) **(Food fortification)**	Góral and Pankiewicz (2017), Góral et al. (2019), U. Pankiewicz et al. (2020)
MRS	*Lb. delbrueckii* subsp. *Bulgaricus CFL1*	60–428 V/cm	Reduced acidification activity: possible application for low acid products	Peng et al. (2020)
Reconstituted skim milk	*S. thermophilus DIL 5218* *Lb. delbrueckii* subsp. *Bulgaricus DSMZ 20081*	Electric field Strength 3.67 kV/cm frequency: 0.5–4 Hz	Enhanced proteolytic phenotype Enhanced oxidative stress response	Chanos et al. (2020)
M17 and chemically defined medium	*L. lactis* subsp. *cremoris*	Moderate PEF	Enhanced EPS production One‐pass treatment (32% increased EPS) Circular treatment (94% increased EPS) Increased cell permeability (7%–10%)	Ohba et al. (2016)
Soymilk	*Lb. casei BT 1088 and BT8633* *Lb. fermentum BT 8219* *Lb. gasseri FTDC 8131*	Field strengths: 2.5, 5.0, and 7.5 kV/cm Pulse durations: 3, 3.5, and 4 ms	Enhanced β‐glucosidase activity (up to 94.6% and 80.4% increase for *L. casei* BT 1088 at 5.0 and 7.5 kV/cm) and isoflavone bioconversion (up to 25%) for food fortification	Ewe et al. (2012)
Air‐dried goose meat	*Lc. plantarum PDD‐1 and L. lactis* subsp. *lactis JCM5805*	Field strengths: 0.7 kV/cm pulse durations: 150 s	– Enhanced flavor and overall quality – Increased activity of monoamine oxidase (232.57 U/mg protein), decreased TBARS (0.3 mg/kg), and TVB‐N (15.75 mg/100 g) content	Y. Zhou et al. (2025)
Watermelon Juice	*Lb. plantarum DSM 9843*	Nano second PEF (electric field strength—0–60 kV/cm, repetition frequency—1–50 Hz, pulse width—35 ns)	Increased production of metabolites l‐Lactic acid (19%) and acetic acid (15%)	Kanafusa et al. (2021)
Chemically defined medium	*S. cerevisiae*	Electric field strength—50–3000 V, time—20 min, pulse width—10–150 µs	Enhanced iron (48.01 mg/g), magnesium (3.98 mg/g), and zinc (15.57 mg/g) uptake **(Food fortification)**	Nowosad et al. (2021), U. Pankiewicz and Jamroz (2010), U. Pankiewicz and Jamroz (2011)
Synthetic fermentation medium	*S. cerevisiae Actiflore F33*	EFS: 20–2000 V/cm *n*: 1–10,000 t PEF: 10–5 to 1 s	– Higher fructose consumption (≈2.33 times for *E* = 100 V/cm and by ≈3.98 for *E* = 6000 V/cm) – Increased extraction of ionic compounds	Mattar et al. (2014) and Mattar et al. (2015)
**Cold plasma, lysozyme and SC‐CO_2_**				
Potato dextrose broth	*Pleurotus ostreatus CGMCC 5.374*	Low‐vacuum cold plasma	Enhanced polysaccharide (3.16%) synthesis	Guo et al. (2024)
MRS	*Lb. acidophilus PIN7*	LYSOZYME Treatment	Reduced DSS‐induced colitis via TLR6 signaling and gut microbiota modulation.	Kye et al. (2022)
Cysteine supplemented MRS	*Bifidobacterium* spp.	SC‐CO_2_	Extraction of polar liquids (2.80 and 3.11 mg of glycolipids, 1.86 and 1.88 mg of phospholipids from *B. longum* and *B. angulatum*, respectively)	Izhyk et al. (2012)
MRS	*Lb. plantarum B‐01*	SC‐CO_2_	Glycolipid extraction (620 µg of glycolipids and 875 µg of phospholipids)	Rakhuba et al. (2009)
**Irradiation**				
MRS	*Lc. casei, Lb. acidophilus, Lc. plantarum*, and *Lc. paracasei*	γ‐Irradiation	Paraprobiotics production (vaccine adjuvants): 2.5‐fold downregulation of IFNα by *Lc. casei* 1‐fold upregulation of IL‐6 *Lc. casei*	Porfiri et al. (2022)
ND	*B. amyloliquefaciens FPTB16* *B. subtilis FPTB13*	UV light (for 2.5 h)	Immunostimulant	Kamilya et al. (2015)
MRS	*Limosilactobacillus reuteri*	γ‐Irradiation (8.05 Gy/min with cobalt 60 for 20 h)	Improved visceral pain in colorectal distension	Kamiya et al. (2006)
ND	*Lb. rhamnosus GG (LGG)*	39‐W germicidal UV lamp for 5 min	Reduced IL‐8 production (59%)	Lopez et al. (2008)
MRS	*Lb. rhamnosus*	120‐min exposure to ultraviolet (UV) light	Enhanced immunobiotic properties	Salva et al. (2021)
Sourdough	*Lc. plantarum LP1, LP25, Pediococcus pentosaceus PP18*	NA	Protects against high‐fat diet‐induced gut damage	Y. Yu et al. (2024)
Durum Wheat Pasta	*Bifidobacterium animalis* subsp. *Lactis 12*	γ‐Irradiation	Reduced glucose by ∼20 mg/dL and total cholesterol by ∼10 mg/dL, and gut microbiota modulation in paraprobiotics fed rats	Almada, Almada‐Érix, Costa, et al. (2021)
**High‐pressure processing**				
PBS	*Lb. rhamnosus* ATCC 53103	High hydrostatic pressure, 400 MPa	Irreversible damage of the membrane. A 7.5 log reduction was achieved	Ananta and Knorr (2009))
PBS	*Lc. plantarum* MIUG BL21	High pressure, 600 MPa, 10 min	Cytocompatibility and antiproliferative effect (172.50% cell viability in comparison to 92.39% of heat treated)	Stănciuc et al. (2024)
Kimchi	*Lb. plantarum* K8	High pressure, 27,000 psi	Prepared parabiotic used as functional ingredient in moisturizing products	H. Kim et al. (2020)

Abbreviations: MRS, de Man, Rogosa and Sharpe broth; ND, not disclosed; PBS, phosphate‐buffered saline; PEFs, pulsed electric fields.

Studies have demonstrated its capacity to enhance the yield of postbiotic components, including biosurfactants and EPs (Hashemi et al. 2022). For instance, Behzadnia et al. (2022) showed that applying ultrasonication at specific stages of fermentation significantly reduced surface tension values, indicating improved biosurfactant yields. Additionally, this process improved antiviral properties during the production of postbiotics from *Lc. plantarum* using agro‐industrial waste such as molasses and whey permeate, highlighting the dual achievement of valorizing low‐cost substrates while enhancing postbiotic production and extraction. Similarly, Chávez‐Alzaga et al. (2024) showed that high‐intensity ultrasonication (HIU) and ultra‐thermosonication (UTS) before fermentation of kefir increased the total protein content while keeping the bioactive properties of produced kefir like control in HIU treatment and obtained a higher level of microbial inactivation in UTS‐treated probiotics. These findings underscore ultrasonication's dual role in advancing both the sustainability of substrate use and the functional performance of postbiotic products. However, the application of ultrasound must be precisely timed; evidence from Behzadnia et al. (2022) indicates that interventions during the 12th hour of fermentation yielded optimal metabolite extraction compared to earlier or later applications. This emphasizes the necessity for rigorous process calibration to maximize efficiency while safeguarding the quality of the postbiotics produced. In addition to enhanced postbiotic production, high‐intensity ultrasound, for example, has also proven effective in enhancing the bioactive and sensory qualities of dairy postbiotics, making these products more appealing to consumers (Barros et al. 2020).

Nascimento et al. (2025) investigated the *Lc. casei* NRRL B‐442 postbiotic production using sonication technology (operating parameters: 3300 W/L, 60–65°C, 10 min) and thermal heat treatment (at 80°C, 10 min). The authors observed increased total polyphenols and ascorbic content in sonicated samples compared to thermal treatment. Postbiotics produces using US treatment outperformed the thermal treatment in terms of their antibacterial activity (65%) against *Escherichia coli* in comparison to 51% inactivation observed in thermally produced postbiotic samples. In another study, ultrasound treatment at operating parameters of power: 792 W per cm^2^ and frequency: 20 kHz, resulted in higher postbiotic production in comparison to thermal method largely due to greater damage to the bacterial cells for the release of postbiotic compounds from *Lb. acidophilus* (LA5), *B. animalis* subsp. *lactis* (Bb‐12) (Almada, Almada‐Érix, Bonatto, et al. 2021; Almada, Almada‐Érix, Roquetto, et al., 2021). Further, *Lacticaseibacillus paracasei B1* and *Lc. plantarum* O24 postbiotics were prepared by using sonication (500 W; 2.5 kHz), resulting in enhanced extraction of CFS with improved antimicrobial and antioxidant properties (Kęska et al. 2025). In a similar kind of investigation, *Lb. helveticus* PTCC 1332 postbiotic products, such as protease enzymes (39.28%), peptides (45.27%), and antioxidant and radical scavenging activity, were increased in sonicated samples (100 W, 30 kHz, 25%–75% amplitudes for 30 min) compared to non‐sonicated samples (Hashemi and Gholamhosseinpour 2020).

Ultrasonication accelerates processing times and reduces energy consumption, addressing critical operational challenges in large‐scale production. These attributes not only enhance the cost‐efficiency of industrial processes but also support the development of novel applications in plant‐based alternatives, beverages, and other functional foods (Ashrafudoulla et al. 2023).

Empirical studies have demonstrated that ultrasonication, when applied strategically during fermentation processes, significantly enhances bioactive metabolite yields and lowers surface tension values, as observed in biosurfactant‐rich postbiotics derived from agro‐industrial waste substrates (Behzadnia et al. 2022). These findings underscore the potential of ultrasound to optimize the recovery of high‐value components, providing a notable improvement over conventional extraction techniques while addressing sustainability goals.

The ability of ultrasound technology to preserve heat‐sensitive bioactive compounds while avoiding the degradation of nutritional and sensory qualities represents a core advantage over thermal processing methods. Traditional heat‐based techniques, though widely used, frequently result in the denaturation or loss of critical postbiotic molecules, including bacteriocins, organic acids, and EPs, thereby compromising the bioactivity of the resulting products (Riza Fathima et al. 2024). In contrast, ultrasound‐assisted processes maintain the structural and functional integrity of these compounds, ensuring that their health‐promoting properties are retained. This preservation is particularly crucial in consumer‐facing functional food products, where maintaining sensory attributes such as flavor, color, and texture is a priority for market acceptance (Prithviraj et al. 2021; Radhakrishnan et al. 2023). Furthermore, ultrasound processing avoids the generation of undesirable thermal byproducts such as furans, which are not only detrimental to product safety but also inconsistent with clean‐label and minimally processed food trends (Radhakrishnan et al. 2023). These features make ultrasound technology an attractive option for developing postbiotics that align with consumer demands for high‐quality, health‐promoting foods.

Recent research has highlighted the superior extraction efficiency of ultrasound technology, particularly in the recovery of phenolic and other bioactive compounds. For example, studies have reported a more than 50% increase in phenolic extraction due to ultrasonic processing compared to untreated controls, illustrating its efficacy in liberating antioxidant‐rich fractions from microbial and plant sources (Morata et al. 2021). Enhanced extraction of metabolites, such as flavonoids, organic acids, and bioactive peptides, is directly associated with elevated anti‐oxidant capacities, which are integral to the health benefits of postbiotics, including their anti‐inflammatory and gut barrier‐enhancing properties. The improved yield and functional properties of these compounds underscore the potential value of ultrasound technology for producing potent postbiotics as well with broad‐spectrum bioactivities. Furthermore, the ability to extract these compounds from fermentation broths and complex matrices demonstrates the versatility and scalability of ultrasound for industrial applications.

The integration of ultrasound processing with fermentation techniques represents an advanced approach to tailoring postbiotic profiles for specific applications. Notably, the use of ultrasound at critical fermentation stages, such as the 12th hour for biosurfactant production, has been shown to improve functional characteristics, including antiviral activities against pathogens like the Newcastle disease virus (Behzadnia et al. 2022). By modulating the extent of cell wall disruption and the release of intracellular metabolites, ultrasound facilitates the production of postbiotics with enhanced bioactivities and targeted health benefits. Additionally, this method supports the valorization of agro‐industrial byproducts as substrates, contributing to sustainability objectives by reducing food waste while creating high‐value functional ingredients. Research into the synergistic effects of ultrasound and fermentation optimization could further advance the development of innovative postbiotics, providing opportunities for customized formulations that address specific nutritional or therapeutic needs (Behzadnia et al. 2022).

The environmental and economic advantages of ultrasound technology are particularly relevant for its industrial‐scale adoption. As a nonthermal method, ultrasound operates at ambient or sub‐ambient temperatures, leading to significantly lower energy consumption compared to traditional thermal processes (Radhakrishnan et al. 2023). Its rapid and targeted microbial disruption reduces the need for additional sterilization or purification steps, further minimizing resource use, including water and cleaning chemicals. The lower energy and resource demand not only reduce greenhouse gas emissions but also lower operational costs, making ultrasound an attractive option for large‐scale production. Furthermore, the alignment of ultrasound processing with circular economy principles, particularly through the utilization of food industry waste streams, reinforces its role in promoting sustainability in the functional food sector. Its potential to address both economic and environmental goals positions as a compelling choice for producers aiming to meet global sustainability targets while maintaining high production efficiency.

The compliance of ultrasound processing with the contemporary definition of postbiotics ensures its relevance in regulatory and market contexts. According to ISAPP, postbiotics are defined as inanimate microbial biomass or their components that confer health benefits (Salminen et al. 2021a). Ultrasound technology effectively inactivates microorganisms while preserving their structural configurations and bioactive properties, producing postbiotics that meet these definitional criteria. This capacity for standardization and consistency is essential for regulatory acceptance and accurate labeling, supporting manufacturers in substantiating health claims and maintaining consumer trust. Furthermore, the production of clearly defined, high‐quality postbiotics through ultrasound processing aligns with evolving regulatory frameworks for novel foods, facilitating international market entry and bolstering the credibility of functional food products.

The expanding use of ultrasound in postbiotic production not only fulfills sustainability and safety goals but also drives innovation in functional food and nutraceutical development. By preserving bioactive integrity, enhancing extraction efficiency, and contributing to resource sustainability, ultrasound technology addresses critical challenges in the postbiotic production process. Its compatibility with existing food manufacturing systems and its ability to integrate with other nonthermal methods provide additional flexibility for industrial applications. Continued research and refinement of ultrasound processing parameters will be essential to fully realize its potential, enabling the development of postbiotics that meet diverse consumer and clinical needs while supporting global sustainability objectives.

##### 4.2.1.2 High‐Pressure Processing (HPP)

HPP, although it remains underexploited for postbiotic production, is another promising nonthermal technology with immense potential for commercialization in the production of postbiotics, offering significant advantages in preserving heat‐sensitive bioactive compounds and microbial metabolites (Pimentel et al. 2023). Similar to other nonthermal technologies, HPP inactivates microorganisms without extensive thermal degradation, thereby ensuring the retention of nutritional and functional qualities and the intended health benefits of postbiotic products (Pegu and Arya 2023; Radhakrishnan et al. 2023). Unlike conventional heat‐based methods, HPP operates by applying pressures typically ranging from 100 to 600 MPa at ambient or mildly elevated temperatures, effectively inactivating microorganisms while avoiding the loss or denaturation of essential postbiotic molecules, including SCFAs, bacteriocins, and EPs (Asefa et al. 2025; Pegu and Arya 2023).

Tsevdou et al. (2020) applied HPP at varying pressures of 100–400 MPa to investigate the production of *Bifidobacterium bifidum* and *Lb. casei parabiotics* in yogurt above 400 MPa of pressure for at least 10 min of treatment. In another study, Stănciuc et al. (2024) stated that HPP can be applied to parabiotic formation of *Lc. plantarum* MIUG BL21 with enhanced antitumor effects. Similarly, HPP treatment resulted in increased formation of polyphenols and SCFAs in *Lc. plantarum* MIUG BL21 and *Lactiplantibacillus paraplantarum* MIUG BL74 that enhance the anti‐tumor effect (Păcularu‐Burada et al. 2024).

HPP preservation capability has pivotal implications for creating innovative formulations, enabling specific health claims like enhanced gut barrier function and immune modulation (Asefa et al. 2025). Evidence from recent studies also suggests that HPP‐treated products achieve superior microbial safety and bioactivity while avoiding the formation of harmful thermal byproducts such as furans (Gao et al. 2024). For instance, microbial safety targets in vegetable‐based infant formulas were achieved at 400 MPa and 45°C for 15 min without compromising product integrity, highlighting the method's potential for safety and quality (Pasdar et al. 2024). These findings illustrate HPP's dual capacity to meet stringent safety standards and maintain key functional properties, positioning it as an effective alternative to traditional methods in the health food sector (Radhakrishnan et al. 2023).

One of the unique advantages of HPP is its minimal impact on sensory and nutritional properties, ensuring high consumer acceptability (dos Santos Rocha et al. 2022; Song et al. 2022). Unlike thermal methods, HPP prevents the formation of Maillard reaction byproducts, such as acrylamide and 5‐hydroxymethylfurfural, which are significant safety concerns, especially for infants and children (Gao et al. 2024; Pasdar et al. 2024). By preserving texture, color, and flavor in postbiotic‐enriched foods, HPP allows manufacturers to cater to consumer preferences for products that combine health benefits with desirable sensory qualities (Radhakrishnan et al. 2023). This is further supported by its ability to maintain the fresh‐like characteristics of foods, a feature highly valued in functional and health‐oriented product markets (Pegu and Arya 2023). Moreover, the nonthermal nature of HPP makes it compatible with clean‐label and minimal processing movements, trends increasingly favored by modern consumers who associate minimally processed foods with higher authenticity and health benefits (Radhakrishnan et al. 2023).

Furthermore, HPP can enable the stabilization of postbiotic metabolites such as SCFAs, bacteriocins, and EPs by preserving microbial cell integrity until strategically targeted lysis occurs (Danaeifar 2022; Rufino Vieira et al. 2024). This controlled release can boost the yield and bioactivity of key postbiotic compounds, facilitating their use in health‐promoting and biotherapeutic applications (Asefa et al. 2025; Zavišić et al. 2024). The timing of HPP treatment in the postbiotic production workflow can also be optimized, for example, by applying the process post‐fermentation but before complete inactivation, to maximize both the quality and quantity of bioactive compounds produced (Zavišić et al. 2024). Such optimization can lead to postbiotic formulations enriched with compounds like lactic acid, acetate, and cyclic dipeptides, known for their antimicrobial and health‐promoting benefits. This capability makes HPP a valuable tool for developing targeted postbiotic profiles with specific applications in health and food preservation (Zavišić et al. 2024) (Table 2). Additionally, HPP conditions can also be standardized to enhance postbiotic extraction and functionality when used with specific microbial strains or novel fermentation matrices, extending its versatility across diverse production systems (Balasubramaniam 2021). Studies reveal that microbial strains like *Lactobacillus* and *Bifidobacterium* respond uniquely to HPP, influencing the yield and bioactivity of derived postbiotics (Ananta and Knorr 2009; Braschi et al. 2021). This dynamic provides opportunities for product differentiation and targeted functionality, offering tremendous potential for creating innovative postbiotic‐enriched formulations using HPP technology. Moreover, the already existing infrastructure of HPP in the food industry further accelerates its integration into industrial workflows, making it a scalable and efficient solution for modern food production (H.‐W. Huang et al. 2017). Despite its tremendous potential in postbiotic production, research evidence related to postbiotic production using HPP is limited, and therefore, collaboration among researchers, industry professionals, and regulators will be crucial for advancing HPP‐treated postbiotics from laboratory‐scale innovation to widespread commercial use (Balasubramaniam 2021; Pimentel et al. 2023). This interdisciplinary approach will help producers and regulators to ensure that consumer trust is maintained while achieving scientifically validated health outcomes, paving the way for more accessible and effective functional foods (Salminen et al. 2021a). By addressing current gaps, HPP can fully realize its potential as a transformative technology in postbiotic production.

##### 4.2.1.3 Pulsed Electric Fields (PEF)

PEF is a very efficient nonthermal method that works as a minimally invasive technique owing to its precision in balancing cell membrane permeabilization and postbiotic production (Y. Zhou et al. 2025). PEF operates through the application of intense electric pulses that create temporary pores in microbial cell membranes, allowing intracellular compounds to be extracted selectively. Therefore, this method facilitates the extraction of intracellular bioactive compounds, such as proteins, EPs, and microbial metabolites, without inducing extensive molecular damage often associated with conventional thermal techniques (Kanafusa et al. 2021). Experimental studies, such as those involving *Lc. rhamnosus, L. paracasei*, and *Saccharomyces cerevisiae*, have demonstrated PEF's capacity to release valuable bioactive substances while preserving their structural and functional integrity (Djukić‐Vuković et al. 2021; Nowosad et al. 2021) (Table 2). Further, PEF conditions, such as electric field strength, pulse duration, and treatment frequency, can be optimized to maximize the release of intracellular metabolites for specific microbial strains and desired postbiotic profiles, all while maintaining cell viability until inactivation is required. Such adjustments pave the way for tailored postbiotic production processes, ensuring higher yields and improved specific functional or health‐related bioactivities (Y. Zhou et al. 2025). For instance, sublethal PEF treatments have been shown to enhance metabolic activity, as a 10% increase in lactic acid production was observed in sub lethally treated *L. rhamnosus* (Djukić‐Vuković et al. 2021). Similarly, higher β‐glucosidase enzyme activity has been observed after PEF treatment among *Lb. casei BT 1088 and BT8633, Lb. fermentum BT 8219*, and *Lb. gasseri FTDC 8131* (Ewe et al. 2012). In other studies, PEF treatment of *S. cerevisiae Actiflore* F33 with PEF has enhanced the extraction of ionic compounds (Mattar et al. 2014, 2015). PEF treatment at 10–24 kV/cm, 110–115 kJ/L, and 80–522 µs is applied to produce inactivated *Lb. plantarum* at 5‐log reduction, the increase in field strength resulted in higher electroporation (Thamsuaidee et al. 2024). Enhanced antibiotic susceptibility was observed for PEF inactivated cells of *Lb. acidophilus* to PEF at 23.5 kV cm^−1^, 1 Hz frequency (Martens et al. 2020). PEF treatment was carried out by controlling the pulse voltage (8 kV/cm) and cycle at 1 µs to study the extent of EPs extraction from *Lactococcus lactis* subsp. *cremoris*. It was observed that the EPs yielded double compared to the untreated samples due to electroporation, which stimulated the EPS metabolism (Ohba et al. 2017). While, in another study an observed 94% increase in EPS production from *Lactococcus. lactis* subsp. *cremoris* fermentation after PEF treatment was observed (Ohba et al. 2016).

Another compelling advantage of PEF is its ability to outperform traditional thermal methods in preserving the structural and functional integrity of thermally sensitive bioactive compounds (Kanafusa et al. 2021; Nowosad et al. 2021). Research has confirmed that PEF‐treated samples exhibit higher bioactive compound yields and stronger bioactivity compared to those subjected to heat‐based processes (López‐Gámez et al. 2021). Additionally, the faster processing times associated with PEF, which typically ranges from nano to microseconds, minimize the risks of thermal degradation, making the technology especially well‐suited for producing heat labile health‐related food constituents.

In addition, the possibility of faster industrial scalability and its economic efficiency also makes PEF more attractive as a nonthermal technology for postbiotic production (White et al. 2025). The continuous processing nature of PEF has been successfully demonstrated in sectors such as the beverage and wine industries. These applications showcase its ability to inactivate spoilage microorganisms while maintaining the nutritional, sensory, and structural qualities of the product (F. V. M. Silva and van Wyk 2021). Such compatibility with industrial‐scale production lines, faster industrial scalability, and economic efficiency ensures that PEF can meet the high throughput demands of food manufacturing, thereby reducing energy consumption and operational costs (Yan et al. 2025). Furthermore, the successful implementation of PEF in these industries illustrates its potential for broader applications, including dairy and juice‐based postbiotic products. Its capacity to maintain product quality while ensuring microbial inactivation positions the technology as a practical choice for large‐scale production (S. Wang et al. 2025).

Another significant feature of PEF is its alignment with evolving regulatory and definitional standards for postbiotics, which PEF meets by ensuring the complete inactivation of microbial cells while preserving their functional metabolites (Barros et al. 2024; Salminen et al. 2021a). This reproducible and precise inactivation capability not only facilitates compliance with safety and labeling regulations but also supports quality assurance. Such consistency is essential for building consumer trust and ensuring the accurate classification of postbiotic products, particularly as regulatory frameworks around postbiotics continue to evolve (Yan et al. 2025).

On the other side, the integration of PEF with other nonthermal technologies offers additional opportunities to enhance postbiotic production. Studies have investigated the synergy between PEF and ultrasound, revealing that their complementary mechanisms—PEF for targeted permeabilization and ultrasound for mechanical cell disruption—lead to improved metabolite yields and functional properties (Kumari et al. 2018). For instance, PEF treatment combined with high‐pressure disruption of *S. cerevisiae* cells showed efficient and selective extraction of different intracellular components such as proteins and ionic components (D. Liu et al. 2013). Similar results were also described by Berzosa et al. (2023), where PEF treatment of yeast biomass resulted in cost‐effective sequential extraction of several value‐added biomolecules such as β‐glucans. These findings highlight the potential for hybrid technologies to optimize resource use, reduce processing times, and minimize the environmental impact of large‐scale production processes (Radhakrishnan et al. 2023). Further exploration of such synergistic approaches is therefore essential to fully realize the combined potential and benefits of these advanced technologies in both functional food manufacturing and biotherapeutic applications. In summary, continuing to refine PEF integration with other technologies and addressing regulatory considerations can play a transformative role in advancing the field of postbiotics and functional foods.

#### 4.2.2 Alternative Processing

Alternative nonthermal processing methods, such as cold plasma, supercritical CO_2_, UV radiation, and ionizing radiation (Figure 2), have also drawn considerable attention for postbiotic production due to their capacity to achieve effective microbial inactivation while preserving the functional and nutritional integrity of bioactive compounds (Pimentel et al. 2023; Zhong et al. 2024; Żółkiewicz et al. 2020). Unlike traditional thermal methods, these technologies provide targeted microbial control without compromising the structural properties of postbiotic components (Suthar et al. 2025). Similar to physical nonthermal methods, these techniques also maintain and may even enhance the bioactivity and stability of key postbiotic metabolites, such as SCFAs and EPs, thereby expanding their utility in the development of functional food products (Chacha et al. 2021). However, despite their promising attributes, the adoption of these methods requires careful examination of their scalability, economic feasibility, compatibility with diverse production matrices, consumer acceptability, need for specialized equipment and precise calibration to avoid overexposure, and present challenges for widespread industrial adoption of alternative nonthermal processing methods for postbiotic production (Hernández‐Hernández et al. 2019; Leong et al. 2024). In addition, initial investments in specialized equipment and personnel training raise doubts about the economic accessibility of these methods for small‐ and medium‐sized enterprises, particularly in less developed regions.

Cold plasma is one such particularly promising technique in postbiotic production due to their dual ability to effectively inactivate microorganisms and selectively preserve or enhance bioactive molecule concentrations (Chacha et al. 2021; de Lima et al. 2022). Emerging evidences have also highlighted the potential of integrating alternative nonthermal processing methods with precision fermentation designs and substrates to create postbiotic formulations with unique functional properties (White et al. 2025). Specific combinations of fermentation techniques with alternative nonthermal interventions have been shown to enrich the bioactive profiles of produced postbiotics, enhancing their anti‐microbial, anti‐oxidant, and anti‐inflammatory activities (Riza Fathima et al. 2024).

Overall, alternative nonthermal processing methods present a transformative opportunity for postbiotic production, offering superior bioactive preservation, enhanced product stability, and added sustainability benefits compared to traditional thermal approaches. However, overcoming the challenges of standardization, scalability, and economic feasibility will be crucial to realizing their full industrial potential. The sections below introduce these cutting‐edge techniques in detail.

##### 4.2.2.1 Cold Plasma Treatment

Cold plasma treatment represents an advanced nonthermal technology that has gained attention for its potential in microbial inactivation while preserving the integrity of sensitive bioactive components (D. Mehta and Yadav 2022; Thirumdas et al. 2015). The mechanism by which cold plasma achieves microbial inactivation is centered on the action of reactive species such as oxygen and nitrogen that penetrate microbial cell membranes, leading to intracellular disruption and eventual cell death without the thermal degradation associated with conventional heat‐based processes (Hassoun et al. 2020). By maintaining the structural and functional integrity of temperature‐sensitive postbiotic compounds such as SCFAs and bacteriocins, cold plasma processing can facilitates the production of high‐quality postbiotics with preserved health‐promoting properties (Ahmadian et al. 2023; Suthar et al. 2025).

Studies have demonstrated the ability of cold plasma treatment to preserve essential metabolites critical for the efficacy of postbiotics, including SCFAs and EPs (Guo et al. 2024). This preservation capacity makes cold plasma as a versatile technology, capable of enhancing both the safety and efficacy of postbiotic‐enriched products, and is particularly advantageous for producing functional ingredients where the retention of bioactivity is paramount (Hassoun et al. 2020).

In comparison to traditional thermal processes, cold plasma's nonthermal approach significantly minimizes the degradation of critical nutrients, such as vitamins, peptides, and polysaccharides, which are often negatively impacted by heat (B. Zhang et al. 2022). The avoidance of such degradation supports the production of postbiotic products with enhanced bioactivity and functional value and thus underlines the capacity of cold plasma to meet the dual goals of safety and efficacy in postbiotic production (Balthazar et al. 2022).

Experimental evidence supports the ability of cold plasma‐treated products to retain microbiological safety while maintaining high levels of bioactive components (Niedźwiedź et al. 2020; Varilla et al. 2020). This dual functionality establishes cold plasma as a reliable choice for developing products that meet regulatory safety standards and functional health benefits simultaneously. Technology's ability to enhance microbiological safety without impairing the nutritional or sensory profile strengthens its application in both food safety and value‐added health product development (H. Liu et al. 2022; Smet et al. 2019). Furthermore, comparative studies indicate that cold plasma treatment has a less detrimental effect on sensory attributes such as color, texture, and flavor than traditional high‐heat methods (Zhao et al. 2020). This capability supports ongoing trends in the food industry, where the preference for natural and minimally processed options plays a key role in shaping purchasing behaviors (Harikrishna et al. 2023).

The industrial scalability of cold plasma is supported by advancements in plasma device technologies, including modular and continuous flow systems, which facilitate integration into high‐throughput manufacturing environments (Harikrishna et al. 2023). However, variability in device configurations, plasma sources, and operating parameters presents challenges that require systematic optimization and standardization (Cassani et al. 2022).

Furthermore, compatibility of cold plasma treatment with other biotechnological approaches, such as fermentation, enables the development of innovative postbiotic formulations designed to target specific health needs or consumer demographics (Dong et al. 2021). Through its capacity to support sustainable, safe, and high‐quality production processes, cold plasma represents a transformative technology for the functional food and nutraceutical sectors. By leveraging its multidisciplinary potential, industries can develop innovative solutions that deliver health‐promoting postbiotics with broad consumer appeal and regulatory compliance. However, further research into the interaction between various plasma species, microbial strains, and food matrices is crucial to developing best practices that ensure consistent outcomes in postbiotic production (Balthazar et al. 2022; Barros et al. 2020).

##### 4.2.2.2 Irradiation

Irradiation (UV radiation, γ‐irradiation, and pulsed light) plays a significant role in probiotic and postbiotic food production, serving as a method to enhance safety and extend shelf life without leaving harmful chemical residues or adversely affecting sensory and nutritional properties (Dong et al. 2021; Kamilya et al. 2015; Salva et al. 2021). The application of irradiation technology in food processing has gained attention due to its ability to effectively reduce microbial contamination without the need for heat treatment, which can often compromise the nutritional and sensory qualities of food products.

In postbiotic production, irradiation can be employed to inactivate live microorganisms, leaving behind their beneficial metabolites and cellular components. These methods leverage electromagnetic radiation to disrupt microbial DNA, achieving high levels of microbial inactivation (Y. Yu et al. 2024). Irradiation can effectively terminate microbial activity while preserving the structural integrity of bioactive compounds, such as SCFAs, enzymes, and peptides, which are responsible for the postbiotic effects (Almada, Almada‐Érix, Bonatto, et al. 2021; Almada, Almada‐Érix, Costa, et al. 2021; Almada, Almada‐Érix, Roquetto, et al., 2021). Therefore, irradiation technologies in combination with some cell lysis methods are particularly advantageous for functional food systems, where consumer preferences increasingly favor minimally processed, clean‐label products. Research has demonstrated the efficacy of UV and pulsed light treatments in maintaining the quality and safety of postbiotic‐enriched products while avoiding the thermal or oxidative damage often associated with conventional methods (Nonglait et al. 2022).

The controlled application of irradiation allows for the reduction of microbial load without significantly altering the nutritional and sensory qualities of the food product (Odueke et al. 2016). Moreover, irradiation can also maintain the freshness and quality of probiotic and postbiotic foods, potentially extending their shelf life and reducing food waste (Shahbaz et al. 2016).

However, careful consideration must be given to the irradiation dose, as excessive exposure may alter the structure of postbiotic compounds and other nutrients (Indiarto et al. 2023; J. Yang et al. 2024). Determining the optimal irradiation parameters is crucial to strike a balance between microbial safety and the preservation of beneficial components. Factors such as the type of food matrix, target microorganisms, and desired shelf life must be considered when designing irradiation protocols for postbiotic products (Gómez‐López et al. 2022). Moreover, the use of irradiation in food processing is subject to regulatory oversight to ensure consumer safety. Different countries have varying regulations regarding the application of irradiation technology and the labeling of irradiated foods (Morehouse 2002; Roberts 2016). Manufacturers have to strictly comply with these regulations and provide transparent information to consumers about the use of irradiation in their products (Y. Zhang et al. 2024).

Ongoing research aims to optimize irradiation protocols to maximize the benefits for postbiotic food production while ensuring product safety and quality. Scientists are exploring innovative approaches, such as combining irradiation with other techniques to create novel postbiotic compounds or enhance the bioavailability of existing ones (Almada, Almada‐Érix, Costa, et al. 2021; Porfiri et al. 2022; Salva et al. 2021). By carefully controlling the irradiation process, it may be possible to induce beneficial modifications in microbial metabolites, potentially leading to improved health outcomes for consumers. These advancements could further enhance the efficacy and applicability of irradiation in the functional food industry.

As the demand for functional foods continues to grow, irradiation technology is likely to play an increasingly important role in ensuring the safety, quality, and efficacy of these functional food products (J. Yang et al. 2024). Therefore, continued research and development in this field will contribute to the advancement of food processing techniques and the expansion of the probiotic and postbiotic market.

##### 4.2.2.3 Supercritical CO_2_

Supercritical CO_2_ (Sc‐CO_2_) treatment has emerged as a promising technique in postbiotic‐production, offering several advantages over conventional methods. This innovative process involves subjecting microbial cells to high‐pressure CO_2_ in its supercritical state, which induces cell disruption and enhances the release of intracellular components (Veiga et al. 2024). The mechanism of action primarily relies on the ability of Sc‐CO_2_ to penetrate cell membranes, causing rapid depressurization and subsequent cell lysis (O'Sullivan et al. 2022). The unique properties of Sc‐CO_2_ make it an ideal medium for postbiotic production. Its low viscosity and high diffusivity enable it to penetrate cellular structures effectively, whereas its low surface tension allows for easy removal from the final product (Izhyk et al. 2012; Rakhuba et al. 2009). This controlled disruption allows for the efficient extraction of bioactive compounds, including peptides, enzymes, and metabolites, which constitute the postbiotic fraction.

Further, the use of Sc‐CO_2_ in postbiotic production offers several environmental and safety advantages as well. CO_2_ is nontoxic, nonflammable, and readily available, making it a safer alternative to organic solvents commonly used in conventional extraction methods (Veiga et al. 2024). Moreover, the process can be conducted at relatively low temperatures, reducing energy consumption and minimizing the risk of thermal degradation of valuable compounds. Additionally, the antimicrobial properties of Sc‐CO_2_ contribute to the preservation of these extracted components, ensuring their stability and bioactivity (Geng et al. 2024). This inherent antimicrobial action helps prevent contamination during the extraction process and extends the shelf life of the resulting postbiotic products. The nonthermal nature of Sc‐CO_2_ treatment also helps maintain the structural integrity and functionality of heat‐sensitive postbiotic components, making it potentially suitable to produce high‐quality postbiotics and products with high sensorial properties (Moreira et al. 2023).

Moreover, the versatility of Sc‐CO_2_ treatment allows for fine‐tuning of process parameters such as pressure, temperature, and exposure time. This adaptability enables researchers and manufacturers to optimize the extraction conditions for specific microbial strains or desired postbiotic components, leading to more targeted and efficient production processes. Besides, the Sc‐CO_2_ treatment method aligns well with the principles of green chemistry and sustainable manufacturing (Amaral et al. 2017). The CO_2_ used in the process can be recycled, reducing waste and environmental impact. This eco‐friendly aspect, combined with its effectiveness, positions Sc‐CO_2_ treatment as a promising technology for the future of functional food, postbiotic production, and nutraceutical industries (W. Wang et al. 2021; I. K. Yu et al. 2018).

As research in this field continues to advance, it is likely that Sc‐CO_2_ treatment will play an increasingly important role in the development of novel postbiotic products with enhanced bioactivity and stability. The potential applications of this technology extend beyond food and nutraceuticals, with possible uses in pharmaceuticals, cosmetics, and other industries where the extraction of bioactive compounds from microbial sources is valuable.

### 4.3 Nonthermal Technologies: Status, Challenges, and Future Prospects in Commercial Postbiotic Production

In terms of nonthermal processing methods employed for postbiotic production, several nonthermal procedures have gained traction in commercial settings. Among these, HPP is currently one of the most commercially advanced nonthermal technologies used in the food industry, including postbiotic production. However, despite its growing adoption, HPP's commercial implementation of HPP in postbiotic manufacturing is somewhat limited by factors such as high capital investment costs and batch‐mode operation, which can affect production and scalability. Nonetheless, the proven effectiveness of this technology in preserving sensory and nutritional quality supports its continued and expanding application in the postbiotic sector, particularly for products requiring gentle microbial inactivation and retention of bioactivity (Balasubramaniam 2021; Shree Kumari and Mohanasrinivasan 2025; P. Yang et al. 2021). Further ahead, ultrasonication is also at the forefront and is increasingly used by companies in the postbiotic production sector to extract intracellular bioactive compounds from microbial cells. Ultrasonication has proven to be valuable for improving fermentation efficiency and enhancing the yield of target postbiotic substances without exposing them to damaging heat (Manyatsi et al. 2024). However, its commercial use is still growing rather than being fully established, as companies continue to optimize parameters such as intensity, duration, and temperature control to balance cell disruption while preserving bioactivity (Taha et al. 2024). Challenges related to scaling up and integrating ultrasonication into continuous industrial processes remain, but ongoing research and industrial interest suggests broader adoption in the near future for efficient postbiotic extraction (Almahbashi and Gunes Altuntas 2025).

Sc‐CO_2_ extraction is favored for obtaining specific postbiotic compounds because it is a green, solvent‐free method that preserves the stability and bioactivity of the sensitive metabolites. However, its use in postbiotic extraction is limited by challenges such as high initial investment costs, complexity of processing heterogeneous microbial matrices, and need for specialized equipment and technical expertise (Riza Fathima et al. 2024; Veiga et al. 2024). Moreover, optimization is required to efficiently extract diverse microbial metabolites, owing to their varying chemical properties and affinities. These factors restrict widespread commercial adoption, although ongoing research aims to overcome these barriers and fully exploit the advantages of supercritical CO2 for selective high‐purity postbiotic recovery.

Scaling up irradiation for postbiotic production can affect both safety and market acceptance in several ways (C. Li et al. 2025; Zhong et al. 2024). From a safety perspective, irradiation effectively inactivates microorganisms without heat, preserving cellular structures and metabolic activity. However, ensuring consistent dosing and uniform exposure on an industrial scale is critical to avoid incomplete inactivation or unintended changes in cellular components that might affect safety or efficacy. From a market acceptance standpoint, irradiation faces challenges owing to consumer perceptions and regulatory labeling requirements (Zhong et al. 2024). Many consumers remain wary of irradiated products because of misconceptions about radiation risks, potentially limiting market penetration. Regulatory authorities also mandate clear labeling of irradiated foods, which can further influence consumers’ purchasing decisions (Meijer et al. 2021). Therefore, efforts are needed to educate consumers about the safety and benefits of irradiation to improve consumer acceptance. Moreover, operational costs and infrastructure requirements for large‐scale irradiation facilities may affect product pricing and competitiveness. Addressing these factors is essential for successful commercial adoption of irradiation in postbiotic manufacturing (Y. Zhang et al. 2024).

PEF and cold plasma technologies are both at an early stage of adoption in postbiotic manufacturing, with ongoing research and pilot applications showing promise, but also exposing significant hurdles. Currently, PEF is used experimentally or in limited pilot‐scale processes to disrupt microbial cells and facilitate the extraction of bioactive compounds from postbiotics (Almahbashi and Gunes Altuntas 2025; Shree Kumari and Mohanasrinivasan 2025). Its appeal lies in its ability to gently inactivate cells and preserve sensitive ingredients, offering continuous processing possibilities. However, commercial use is still rare owing to high equipment costs, the need for process optimization, and the challenge of scaling up while ensuring consistent efficacy and safety across products (Balthazar et al. 2022; Riza Fathima et al. 2024). Standardizing PEF parameters and meeting rigorous regulatory requirements remain unresolved issues that limit its full‐scale adoption at commercial scale. Cold plasma technology is largely experimental, with early‐stage commercial interest in postbiotic production. It is valued for its capacity to inactivate microbes and modify surface properties without causing thermal damage, which may protect or enhance the functional quality of postbiotic ingredients. The push for safe, residue‐free processing has positioned cold plasma as a potential solution for sterilization and bioactive modulation (H. M. Abbas et al. 2024). Nevertheless, current adoption is restricted by the challenge of controlling plasma exposure to optimize antimicrobial effects without damaging beneficial molecules and the difficulty of scaling up continuous industrial production. The cost of equipment, process reproducibility, and lack of established regulatory frameworks are major limiting factors. Overall, nonthermal technologies offer significant future potential as next‐generation methods in postbiotic manufacturing. Advances in engineering, process control, and scientific understanding will likely broaden their commercial application, enabling the safe and efficient production of high‐quality postbiotics. If these challenges can be addressed, nonthermal technologies may help drive innovation, improve process sustainability, and meet the increasing demand for natural and functional ingredients in the food and health sectors (Hernández‐Hernández et al. 2019; Pivarnik and Worobo 2014; A. Silva et al. 2024; Thirumdas et al. 2015; White et al. 2025).

## 5 Industrial Implementation

Optimization of various process parameters and assurance of quality are vital steps in transitioning innovative postbiotic technologies from lab research to industrial use. Overall, factors like process calibration, technological advancement, and stringent quality control support the production of safe, effective, and scalable postbiotic products. These insights are crucial for fully leveraging nonthermal methods within the broader scope of advancing health‐promoting solutions in the food and nutraceutical industry.

### 5.1 Process Optimization

The growing recognition of postbiotics in functional food markets underscores the importance of maintaining product quality and safety standards, which are critical for consumer acceptance and market growth (Balthazar et al. 2022; Pimentel et al. 2023; Salminen et al. 2021a). The transition from laboratory‐scale postbiotic production to industrial environments demands the precise application of optimized parameters and validated concentrations. Although current advancements show promise, further efforts are needed to establish global regulatory frameworks that support the commercialization of postbiotics (Wegh et al. 2019; Wei et al. 2024). The optimization of nonthermal postbiotic production processes involves careful calibration of processing conditions to adapt to the specific characteristics of microbial strains and substrates. Factors such as pressure levels in HPP, electric field strength in PEFs, and frequency, power, and treatment duration during ultrasonication significantly influence postbiotic yield and the bioactivities of the final products (Zhong et al. 2024; Żółkiewicz et al. 2020). A systematic evaluation of different process parameters for nonthermal technologies is therefore imperative in identifying interactions between variables and their impact on microbial fermentation outcomes. This approach will not only facilitate the reproducibility of postbiotic production but will also enable predictive modeling for industrial scalability. Adopting process control strategies not only ensures regulatory compliance but also strengthens consumer trust by consistently delivering safe and effective postbiotic products.

Further, the challenge of ensuring effective microbial inactivation together with the preservation of sensitive bioactive compounds is central to the optimization of nonthermal processes. Nonthermal methods, operating under milder conditions, avoid the degradation of bioactive commonly observed in heat‐based approaches. This balance is essential for producing postbiotics that align with the consensus definition, which requires nonviable microbial cells or their components to confer health benefits (Balthazar et al. 2022; Pimentel et al. 2023; Salminen et al. 2021a). Achieving this dual objective remains a critical area of research, particularly in maintaining consistency between efficacy and regulatory standards.

Another aspect for optimization in postbiotic production efficiency is the dynamic nature of food matrices that require continuous real‐time monitoring and adaptive adjustments to nonthermal processing parameters for ensuring consistency in product quality and addressing batch variability. As this is a common challenge when scaling from laboratory to industrial volumes, implementing such systems will not only reinforce functional and safety benchmarks but will also ensure the stability of postbiotic‐enriched foods during production and storage (Suthar et al. 2025; Zavišić et al. 2024). The development of predictive models based on real‐time monitoring data potentially could offer pathways to mitigate fluctuations in raw material quality or processing conditions. However, the complexity of interactions between processing parameters and microbial responses necessitates the refinement of these models for broader applicability and current limitations in advance monitoring technologies warrant further development for ensuring the high‐throughput, reproducible production of postbiotics (Balthazar et al. 2022; Barros et al. 2024; Bhatia et al. 2024).

The integration of nonthermal technologies into industrial production lines offers scalable and energy‐efficient solutions that align with sustainable manufacturing practices. By lowering operational costs and environmental impacts, these methods provide significant competitive advantages for the food industry, especially as consumer demands for clean‐label and minimally processed products continue to grow (Pasdar et al. 2024; Vera‐Santander et al. 2024; Wei et al. 2024). However, the economic feasibility of implementing such technologies on a large scale requires additional cost‐benefit analyses to ensure their long‐term sustainability.

The strategic selection of nonthermal technologies for specific end‐product characteristics is a key consideration in industrial applications. Economic, regulatory, and environmental factors play a major role in determining the appropriateness of nonthermal technologies for postbiotic production. For example, targeting antimicrobial activity may necessitate PEFs for effective membrane disruption, whereas preserving sensory qualities may favor HPP or ultrasound techniques, both of which retain flavor and texture better than heat‐based alternatives (Balthazar et al. 2022; White et al. 2025). These decisions must be guided by a thorough understanding of each technology's capabilities and limitations to align with production goals. Certain methods offer advantages such as lower energy consumption, reduced greenhouse gas emissions, and straightforward regulatory compliance due to consistent microbial inactivation and product standardization (Thirumdas et al. 2015; W. Wang et al. 2021; White et al. 2025). However, challenges remain in balancing these benefits with the upfront investments required for implementing nonthermal technologies, particularly for smaller manufacturers.

Aligning technological choices with product development goals ensures that advancements in postbiotic research are effectively translated into scalable, market‐ready applications. This alignment supports the functional food sector's evolution toward sustainable, health‐promoting products that meet consumer and regulatory expectations. While promising, achieving this alignment requires further collaboration between researchers, manufacturers, and policymakers to address the challenges of industrial integration and global competitiveness.

### 5.2 Quality Considerations

Quality assurance in nonthermal postbiotic production places significant emphasis on controlling safety parameters to ensure that postbiotics are free from viable and potentially pathogenic microorganisms. HPP and pulsed light have been extensively studied and are recognized for their efficacy in inactivating a wide spectrum of microorganisms, thereby minimizing the risk of viable cell survival in the final product (Ananta and Knorr 2009; Balasubramaniam 2021; Yan et al. 2025). This is particularly relevant for postbiotic formulations, as they are intended to confer health benefits without the potential adverse effects associated with live bacteria. The robust microbial inactivation provided by nonthermal technologies supports their reliability and broader application in functional food products. However, further studies are warranted to evaluate the long‐term safety profiles of postbiotics processed through these methods and how their antimicrobial effects vary across diverse substrates and microbial compositions (Homayouni‐Rad et al. 2025; Pivarnik and Worobo 2014; Rad et al. 2020). Comprehensive safety control measures are indispensable in ensuring that nonthermal postbiotic production processes meet both regulatory and consumer expectations. Regular monitoring of microbial counts, detection of endotoxins, and assessment of other microbial byproducts are critical components of quality control strategies. Moreover, these measures must be harmonized with food‐grade processing standards and the international definitions of postbiotics (Liang et al. 2024). The assurance of safety in these products is particularly noteworthy because their advantages over probiotics—namely, the absence of viable organisms—are only achieved through rigorous oversight during production (Liang et al. 2024; Vinderola et al. 2022b). Further work is needed to standardize detection protocols and ensure their compatibility with a wide range of processing environments, ensuring that small‐scale and industrial producers alike can comply with stringent safety thresholds (Rad et al. 2020; Wei et al. 2024).

Nonthermal technologies significantly enhance the safety of postbiotic production by avoiding the generation of process‐derived contaminants, which are often associated with thermal treatments. For example, HPP, PEFs, and pulsed light do not produce heat‐induced degradation products, aligning with consumer demands for clean‐label and minimally processed foods (Wei et al. 2024). The sustainability aspect of these methods also underscores their ability to reduce the environmental impact of postbiotic production while delivering high‐quality and residue‐free products. Despite these advantages, further comparative studies are essential to evaluate the differences in the type and extent of contaminants across various nonthermal methods and to solidify their status as environmentally friendly alternatives (Almada, Almada‐Érix, Bonatto, et al. 2021; Pivarnik and Worobo 2014; Wei et al. 2024).

Another aspect to be considered in quality assurance of postbiotics is the durability and storage stability of postbiotics produced using nonthermal methods. It is imperative that postbiotics should be able to retain and provide substantial commercial and scientific benefits even under challenging storage conditions (Blazheva et al. 2022). Postbiotics are known to exhibit remarkable resilience to environmental stresses such as temperature fluctuations, humidity, and light exposure, which contrasts starkly with the fragility of live probiotic formulations (Żółkiewicz et al. 2020). This enhanced stability expands the logistical feasibility of integrating postbiotics into various food systems. However, more extensive research is necessary to quantify the exact shelf life improvements afforded by different nonthermal methods and to establish predictive models that correlate specific production conditions with long‐term product stability and efficacy (Sharafi et al. 2024; Wei et al. 2024).

The extended shelf life of nonthermally processed postbiotics directly contributes to their economic and practical viability for integration into a wide variety of food formats. Whether incorporated as dry powders, liquid concentrates, or active packaging components, these products demonstrate minimal degradation under typical distribution and storage conditions (Sharafi et al. 2024). This practical advantage positions postbiotics as superior alternatives to live culture‐based products, which often face logistical challenges due to their sensitivity to external conditions. Nonetheless, more research is required to evaluate the compatibility of postbiotics with complex food matrices to ensure their functional integrity remains consistent during industrial‐scale applications (Sharafi et al. 2024).

The ability of nonthermal processing methods to preserve bioactive properties enables the development of postbiotics with consistent effectiveness and predictable health benefits, fostering consumer confidence and supporting health claims (Balthazar et al. 2022; Hua et al. 2022; Pimentel et al. 2023). By inactivating enzymatic and metabolic degradation processes, nonthermal technologies ensure that bioactive compounds such as bacteriocins, EPs, and organic acids remain intact. However, it remains critical to investigate the extent to which the structural integrity of these molecules is preserved across different processing conditions and to optimize production methods for maximum functionality (Wei et al. 2024).

Quality control protocols in postbiotic production must ensure the integrity and functionality of sensitive bioactive compounds, which are often susceptible to denaturation during thermal treatments. Nonthermal technologies, such as cold plasma and ultrasound, are particularly effective at preserving the structural and functional properties of these molecules, making them ideal for postbiotic production. Analytical techniques to routinely quantify the concentration and bioactivity of functional constituents are crucial for confirming efficacy and ensuring compliance with product specifications. This further emphasizes the need for industry‐standardized methods to validate the retention of antimicrobial and antioxidant properties in diverse food matrices post‐processing (Pivarnik and Worobo 2014; Sharafi et al. 2024).

Continuous assessment is vital to ensure bioactive profiles remain consistent across production batches and scalable volumes from laboratory settings to industrial environments. This consistency supports the regulatory and marketing claims related to postbiotic safety and efficacy (Benkowski et al. 2023; Stelmach et al. 2024). It is essential to develop robust systems that allow for batch‐to‐batch reproducibility while maintaining the functional integrity of the bioactive compounds. However, more research is needed to explore the variability introduced by substrate composition, microbial strain selection, and nonthermal treatment parameters (Pivarnik and Worobo 2014; Wei et al. 2024).

Further to this, regulatory definition and proper labeling of postbiotics play a critical role in enabling market authorization and fostering consumer trust. Compliance with frameworks such as those established by the ISAPP ensures that postbiotics are accurately identified as inanimate microorganisms or their components with proven health benefits (Salminen et al. 2021a; Siciliano et al. 2021; Zavišić et al. 2024). Transparent labeling practices that disclose production methods, microbial sources, and validated health claims are essential for facilitating regulatory reviews and bolstering consumer confidence. However, achieving consistency in regulatory definitions across various markets remains a persistent challenge that must be addressed to promote the global adoption of postbiotics as functional food ingredients (Siciliano et al. 2021; Wei et al. 2024). To this date, the regulatory framework for postbiotics is still emerging and varies across the globe, whereas several nonthermal processes are starting to see adoption by companies, and others remain largely at the research and development stage (Amobonye et al. 2025; Guglielmetti et al. 2025; Prajapati et al. 2023). The regulatory landscape for postbiotics is currently evolving, reflecting the relatively recent emergence of this category within the functional food and pharmaceutical sectors. Unlike probiotics or prebiotics, postbiotics lack universally established and specific regulatory frameworks, which creates both complexity and flexibility for companies seeking to bring products to market (Riza Fathima et al. 2024; Stelmach et al. 2024; Suthar et al. 2025). Presently, regulatory oversight applies according to the product's classification—whether as a food ingredient, dietary supplement, or as pharmaceutical agents. Authorities like the FDA in the United States and European Food Safety Authority (EFSA) in Europe require comprehensive evaluation of safety, identity, purity, and intended health benefits, relying largely on general guidelines established for bioactive compounds and novel food ingredients (Guglielmetti et al. 2025). Manufacturers must comply with good manufacturing practices (GMP), ensure batch‐to‐batch consistency, control contaminants, and conduct risk assessments that demonstrate safety for human consumption. The absence of a dedicated regulatory framework for postbiotics poses challenges for standardization but also encourages rigorous scientific characterization and safety validation, which are critical for regulatory acceptance and consumer confidence globally. Moreover, harmonization efforts and global regulatory dialogues are increasing to develop clear definitions and acceptance criteria for postbiotics, which will facilitate their regulatory approval process and market expansion in the coming years.

Adherence to regulatory definitions is particularly critical for the export and international trade of postbiotics. Variations in definitions or insufficient documentation can severely limit access to global markets (J. E. Aguilar‐Toalá et al. 2021). Aligning with the ISAPP and emerging regional guidelines mitigates these risks and fosters collaboration between researchers, industry professionals, and policymakers. Nevertheless, the establishment of harmonized global definitions and regulatory practices remains an unmet need in the field, potentially hindering the scalability of postbiotic production (Salminen et al. 2021a, 2021b; Wei et al. 2024).

Although significant progress has been made in nonthermal postbiotic production, there are still gaps in harmonized standards, mechanistic understanding, and safety validation. Future efforts must focus on creating universally applicable standard operating procedures and validated analytical methods to enable cross‐study and cross‐industry comparability. Mechanistic research into the action pathways of individual components, such as specific cell wall fragments or secondary metabolites, is also necessary. Elucidating these pathways will not only optimize production processes but also improve the therapeutic index of resulting postbiotic products (Rad et al. 2020; Wei et al. 2024).

Safety assessments must be conducted on a case‐by‐case basis, particularly for novel microbial strains and nontraditional substrates, as unforeseen allergenic or bioactive responses may arise. Further preclinical and clinical trials are required to build a comprehensive safety profile for postbiotics (Benkowski et al. 2023). This includes studies on minimum effective dosages, duration of activity, and potential interactions with other dietary or pharmaceutical compounds to ensure health claims are scientifically substantiated (Rad et al. 2020).

Finally, within the therapeutic context, further research is needed to establish evidence‐based recommendations for integrating postbiotics into preventive and therapeutic programs. Defining clear guidelines for their effective use will enable the development of targeted postbiotic products with specific health functionalities. These efforts will not only support the commercialization of postbiotics but also advance their integration into clinical and functional food applications (Rad et al. 2020; Siciliano et al. 2021).

## 6 Postbiotics Food Applications: Current and Emerging Applications

**FIGURE 3:**
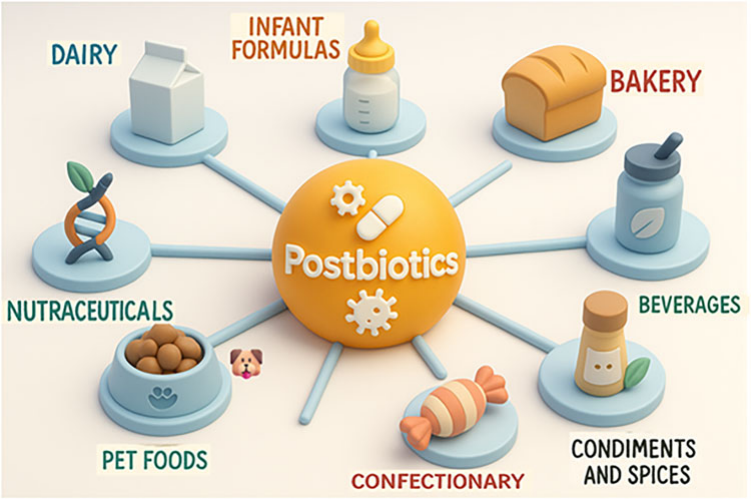
Possible food applications for postbiotics as ingredients.

The postbiotic ingredients market is expected to reach a valuation of USD 16 million by 2025, with projections indicating an increase to USD 45 million by 2035 (Choudhury 2025a). The inclusion of postbiotic ingredients to develop commercial postbiotic food products represents a rapidly growing segment of the functional food market, offering unique health benefits combined with stability and versatility that traditional probiotics often lack (Table 3 and Figure 3) (Żółkiewicz et al. 2020). Unlike probiotics, postbiotics do not require refrigeration, can survive heat processing, and pose no risk of infection or microbial imbalance. Therefore, postbiotics offer a compelling addition to product formulations due to their stability, compatibility with existing food systems, and demonstrate benefits for both physical and mental health. While exploring the options of nonthermal postbiotics in direct food applications, information is primarily scarce. The postbiotics can exhibit their properties in solid or liquid form and can be easily added to the food formulation. Based on their usage, postbiotic food applications can be categorized into enhancing shelf life, antimicrobial and functional packaging, as food additives, food quality improvers, and food supplements (Suthar et al. 2025; Zhong et al. 2024).

**TABLE 3:** Commercially available postbiotic ingredients and their possible food applications.

Product name	Company/Brand	Postbiotic component(s)	Production method	Details and health claims
**Humiome Post LB**	dsm‐firmenich	Inactivated *Lb. delbrueckii* CNCM I‐4831, *Lb. fermentum* CNCM I‐2998	Heat‐inactivation and drying	Derived from two heat‐inactivated strains; supports gut and immune health; stable under harsh processing conditions. Marketed as helping maintain digestive balance and immune modulation
**Postbiotic LB356R**	Lactobio/DKSH	Lysate of *Lactiplantibacillus plantarum*	Freeze‐drying (lyophilization)	A heat‐treated lysate rich in bioactive peptides and metabolites; used in skincare and supplements; claims skin microbiome support and barrier protection
**PoZibio**	Postbiotics Inc./Sabinsa	Heat‐treated *Lb. paracasei*	Heat‐inactivation	Targets leaky gut syndrome, reduces age‐related inflammation, supports cognitive function and promote a healthy gut microbiome
**EF‐2001**	Bereum (South Korea)	Heat‐killed *Enterococcus faecalis* EF‐2001	Heat‐inactivation	Immune enhancement and anti‐inflammatory effects. Extensively researched in human and animal studies
**IMMUSE**	Kyowa Hakko Bio (Kirin)	Heat‐treated *Lactococcus lactis* strain Plasma	Heat‐inactivation	Targets plasmacytoid dendritic cells (pDCs), which are rare immune cells; shown to support innate and adaptive immunity; already being used in multiple supplements and beverages
**Plenibiotic**	Kerry Group	Rice‐derived postbiotic from thermally inactivated *Lacticaseibacillus paracasei* 327	Heat‐inactivation	Gut–skin axis; resilient under processing conditions and stabile shelf life and suitable for functional foods and beverages
**EpiCor**	Cargill	*Saccharomyces cerevisiae* fermentate (EpiCor) containing	Gentle drying	Fermented yeast product proteins, peptides, antioxidants, polyphenols, organic acids, nucleotides, polysaccharides (1–3 1–6, β‐glucans), and mannans rich in metabolites, peptides, beta‐glucans; supports immune function and gut barrier. Have been used in both human and pet supplements
**CoreBiome**	Compound Solutions	Tributyrin (Most powerful SCFA)	Information not Available	Digestive health, female support, gut–brain, gut–heart, gut–muscle, longevity formulas, and weight management
**Total Gut Complex**	Dr. Emil Nutrition	Blend of prebiotics, probiotics, and postbiotics	Heat‐inactivation	Marketed as a comprehensive gut health product; specific postbiotic strain not publicly disclosed. Aimed at consumers seeking “all‐in‐one” gut solutions
**Full Spectrum Postbiotics**	Gaia Herbs	Fermented plant extracts (made with lactic acid bacteria from spontaneous fermentation in either ginger–turmeric, sauerkraut, ginger–beet or kimchi capsules)	Information not Available (extracted without harsh solvents)	Uses traditional fermentation; promotes gut microbial diversity and balance; holistic and herbal‐focused audience. Contains metabolites like organic acids and polyphenol derivatives
**Sauerkraut Postbiotic**	Gaia Herbs	Fermented Sauerkraut juice extract	Information not available	Supports healthy lower GI function by promoting regularity and cleansing with beneficial bacteria
**HT BPL1 Postbiotic**	Archer Daniels Midland Company (ADM) and Biopolis science	Heat treated *Bifidobacterium animalis* sub. *lactis* CECT 8145 and its Lipoteichoic acid	Heat‐inactivation	Reduces waist circumference, visceral fat, and HOMA‐IR scores; ideal for use in food, beverage and dietary supplement formulations

Abbreviation: SCFAs, short‐chain fatty acids.

### 6.1 Functional Food Ingredients

Some food and pharmaceutical industry postbiotic applications relate to dairy products, infant formulas, cereal products, snacks, and functional beverages (Suthar et al. 2025). Dairy‐based foods are among the most prominent targets for postbiotic inclusions, with products like yogurt incorporating postbiotic ingredients to promote digestive and immune health (Sadighbathi et al. 2023). In Asia, fermented drinks Calpis derived probiotic strain *Lb. gasseri* CP2305 was explored for production of postbiotic CP2305 containing heat‐inactivated strains and is reported to possess multiple health benefits such as improvement in gut functions, and sleep quality with relief in stress, anxiety, and mood fluctuations (Chudzik et al. 2021; Sugawara et al. 2019; Toyoda et al. 2020). Similarly, Tetra Pak developed their first postbiotic containing cheese “Fettine Protein+” cheese in collaboration with Inalpi and is already available in markets with cheese slices enriched with postbiotics, selenium, and zinc supporting immune system and overall health (International 2024). Similarly, Danone Nutricia developed a partially fermented infant formula supplemented with postbiotics from, heat‐killed *Bifidobacterium breve*, *Lb. paracasei* along with 3′‐GL, 2′‐FL, and milk fats that act as a doppelganger of breast milk (Szajewska et al. 2022; Vandenplas et al. 2020). Wei et al. (2024) reported that the yeast postbiotic components like vitamins, proteins, phenolic compounds, and organic acids have several food applications. Shigwedha (2014) reported that cell lysate as postbiotics may be added to food and beverages as an ingredient or nutritional supplement with high stability and shelf‐life. Postbiotic *B. animalis* subsp. *lactis* CECT 8145 SCFA was used in the infant slurry formula to study the fat deposition and gut microbiota modulation, and the authors observed a reduction in fat deposition, an increase in SCFA like acetate and lactate and modulated the gut microbiota similar to those of breastfeed infants (Plaza‐Diaz et al. 2023; Ruiz‐Ojeda et al. 2023). Cape gooseberry yogurt enriched with *E. coli* postbiotics observed an increase in organoleptic characteristics like appearance, mouthfeel, and overall acceptability of yogurt except for color (Darwish et al. 2022). Kürşad İncili et al. (2023) analyzed the shelf‐life of the food model by adding a freeze‐dried paraprobiotic of *P. acidilactici* as an antimicrobial agent against food pathogens. Whey‐based grape juice incorporated with *Lc. casei* 01 postbiotics observed a reduction in postprandial glycemia (Barros, Grom, et al. 2021). Tomasik and Tomasik (2020) extracted phytase from *Bifidobacterium longum* spp. *infantis* as a postbiotics to decrease the phytate content in cereal mixture. The authors have also observed an increase in the myoinositol triphosphate. The loss of B‐group vitamins is a common phenomenon during the milling operation of cereals. The lost vitamins can be fortified by adding postbiotics in the cereal mixtures (Tomasik and Tomasik 2020).

Ali et al. (2019) added *Lb. delbrueckii* subsp. *bulgaricus* postbiotic EPs to improve the fermented milk texture and sensory profile. Similarly, lactic acid bacteria EPs are added to bakery products as a food additive to improve dough's rheological and viscoelastic properties (Lynch et al. 2018).

### 6.2 Antimicrobials Applications in Foods

Further, postbiotics have garnered considerable interest in recent years as natural antimicrobial agents that can serve as alternatives or complements to conventional antimicrobial compounds used in food processing. Unlike traditional antimicrobials, such as organic acids, sulfites, nitrates, and synthetic chemicals, which often exhibit broad‐spectrum antimicrobial effects but may pose concerns related to safety, sensory changes, and consumer acceptance, postbiotics often exhibit targeted antimicrobial activity with lower toxicity and reduced risks of resistance development, aligning with the rising consumer demand for clean‐label and natural food preservatives (J. Aguilar‐Toalá et al. 2018; Moradi et al. 2020; Sharafi et al. 2022, 2024). Furthermore, postbiotics also have multifunctional nature that extends beyond antimicrobial action and includes properties such as immunomodulatory, gut microbiome modulation, and antioxidant effects, which may benefit both food safety and human health (Guglielmetti et al. 2025; Mosiej et al. 2025; Suthar et al. 2025). Sharafi et al. (2022) used *Lb. acidophilus* LA‐5 and *B. animalis* BB‐12 postbiotics in the whey media to prepare high‐moisture mozzarella cheese and investigated the antimicrobial and sensory properties. The authors observed a 1.5–2 log reduction of yeast, molds, and bacteria, enhancing the shelf‐life. Egyptian cheese was prepared by incorporating chitosan nanoparticles containing postbiotics produced from different *Lactobacillus* bacterial species to exhibit antibacterial and antifungal properties (Sharaf et al. 2019). As a postbiotic antimicrobial compound, phenylacetic acid can be applied to various food products as an antibacterial and anti‐virulence (Rajanikar et al. 2021). Anti‐*L. monocytogenes* properties of postbiotics of *Lactiplantibacillus sakei* were investigated on beef fillets as a natural preservative (Valipour et al. 2024). Similarly, antimicrobial properties of postbiotics like organic acids and bacteriocins are isolated in *Lb. curvatus* B.67 and *Lb. plantarum* against the *L. monocytogenes* biofilms in food industries (Hossain et al. 2021). As an antifungal agent, soybean grains were preserved using *Lb. plantarum* YML007 CFS (Ahamd Rather et al. 2013). Despite these, postbiotics antimicrobial applications also face several limitations when compared to conventional food antimicrobials. Their antimicrobial spectrum tends to be more strain‐ or species‐specific, requiring careful selection and optimization for effectiveness against particular foodborne pathogens or spoilage organisms (Chang et al. 2021; Ooi et al. 2021). Additionally, postbiotic compounds can be sensitive to environmental conditions such as pH, temperature, and interactions with food matrix components, which may affect their stability and efficacy during food processing and storage (Ebrahimi et al. 2021).

### 6.3 Food Packaging

A few scientific studies have been conducted on using postbiotic components in packaging material, which has several benefits. Yordshahi et al. (2020) developed a nano meat‐wrapping packaging material by incorporating the *Lb. plantarum* postbiotics into the bacterial nano‐cellulose to study the antimicrobial properties. A similar bacterial nano‐cellulose antimicrobial membrane was fabricated against food pathogens for food application (Mohammadi et al. 2022).

### 6.4 Functional Beverages

In other area of postbiotic supplementation potential, functional beverages are another key area where postbiotics have gained commercial traction. In a recent collaboration with AB Biotek, Tetra Pak has announced the introduction of a range of innovative postbiotic food solutions ranging from tea, plant‐based beverages, sports drinks, and more (Pak 2023). Similarly, Kirin's IMMUSE range, using heat‐killed *L. lactis* strain plasma, is integrated into drinks and tablets that target immune enhancement (Thian 2023).

### 6.5 Bakery and Snacks

Further, the application of postbiotics is not limited to refrigerated or liquid foods, because postbiotics can withstand high temperatures baked goods and snack products are beginning to feature postbiotic components as well., companies such as Kerry Group and Cargill are exploring their inclusion in protein bars, powdered snacks, and heat‐treated cereal products (Choudhury 2025a; Morán and Kilasoniya 2024).

### 6.6 Nutraceuticals

Nutraceuticals and supplement blends are another common format, with products like EpiCor by Cargill known for promoting immune and gut health and having the potential to be used for food formulation since it has a long shelf life of 3 years and can withstand a range of pH and is heat stable as well (Inchingolo et al. 2019).

### 6.7 Pet Foods

Postbiotics are even making their way into pet nutrition, specialized formulations for pets now include postbiotics to aid in animal digestive health and overall wellness (Choudhury 2025b; Fritsch and Gross 2021). Manufacturers are continuously innovating and creating new formulations and delivery methods to improve the stability and effectiveness of postbiotic compounds within pet food products. For example, Primal Health's dog chews and EF‐2001‐containing pet foods in Asia, which help support digestive balance and skin integrity. For instance, H&JIN EF‐2001 Premium Probiotic for Cat & Dog contains heat killed *Enterococcus faecalis* and its derived metabolite that are reported to have skin improvement, protect against colitis and fat accumulation in liver, and also enhance vitamin B1 accumulation (Choi et al. 2023). Similarly, EpiCor manufacturers are also claiming that this postbiotic with is anti‐oxidant power and supports for immune defenses can help dogs live more healthy days (Choudhury 2025b).

Despite their many advantages, postbiotic commercial products still face challenges, including limited regulatory recognition and low consumer awareness. Although probiotics are widely understood and accepted, the concept of postbiotics remains relatively new, requiring companies to invest in clear communication and education. Additionally, though early clinical results are promising, more human trials are needed to validate the specific benefits of various postbiotic strains and compounds. Still, the industry is moving swiftly to meet these needs. Major food and ingredient companies, including ADM, Kyowa Hakko, and IFF, are investing in research and development, regulatory approval, and global partnerships to bring postbiotic‐enhanced products to market. These efforts are complemented by a growing consumer appetite for health‐promoting foods, particularly those that offer immune support, digestive balance, and are compatible with busy lifestyles. Postbiotics are uniquely suited to meet these demands due to their safety profile, formulation flexibility, and robust shelf stability. As consumers continue to prioritize health, transparency, and functionality in their food choices, postbiotics are expected to play a central role in the next generation of functional food innovation. With applications expanding from dairy and beverages to snacks, bakeries, supplements, and even pet food, commercial postbiotic products are well‐positioned to transform the landscape of health‐focused food products around the world.

## 7 Regulatory Framework for Postbiotics: Current Status and Emerging Requirements

The regulatory landscape for postbiotics remains fragmented and underdeveloped globally. Unlike established categories such as probiotics, postbiotics currently lack dedicated regulatory frameworks, creating both challenges and opportunities for manufacturers, regulators, and consumers (Guglielmetti et al. 2025; Vinderola et al. 2023). Government authorities worldwide are exploring postbiotic regulation, but till date no official published guidelines or regulations exist specifically for postbiotics as food ingredients or dietary supplements (Amobonye et al. 2025). Instead, postbiotic products are regulated under existing statutes for foods, dietary supplements, or pharmaceuticals, depending on their intended use. The regulatory approach varies significantly across various jurisdictions. For instance, in Japan postbiotic containing foods are being allowed under current frameworks of foods with functional claim; in Thailand, however, various supplements are available that contain postbiotics and have received approval from Thai FDA. Similarly, the United States regulates postbiotic products under its New Dietary Ingredient (NDI) submissions, and Health Canada approves the use of term “postbiotics” in situation where producers provide adequate evidence. Australia's Therapeutic Goods Administration (TGA) has also allowed the inclusion of postbiotic ingredients in Listable Medicines. No regulations could be found for postbiotics in India, China, Brazil, and South America. Similarly, no special regulation framework for postbiotics exists in Europe and has to follow the guidelines associated with food supplements depending on their intended use. At present, Regulatory authorities in China has released industry standards for quantifying postbiotics using flow cytometry and fluorescent quantitative PCR methods (Amobonye et al. 2025; Guglielmetti et al. 2025).

In most of the countries, regulatory authorities require postbiotic production to adhere to GMP, ensuring operations are traceable, facilities meet specifications, and materials are consistent. Comprehensive systems must guarantee the Safety, Quality, Identity, Potency, and Purity (SQuIPP) criteria for manufactured products (Thorakkattu et al. 2022). Essential safety components include implementation of hazard analysis systems to monitor chemical, allergenic, physical, and biological contaminants throughout production. Critical control points (CCPs) must be identified and monitored, with corrective measures implemented as needed. Safety considerations for postbiotics should align with established standards for live microorganisms, as postbiotics originate from microbial parent strains (Thorakkattu et al. 2022). This includes evaluation of antimicrobial resistance genes, virulence factors, and toxin production capabilities. The lack of consensus on postbiotic definitions further adds up to the regulatory uncertainty (Guglielmetti 2023). The divergent terminology and absence of harmonized criteria hinder the development of specific regulatory frameworks. Regulatory authorities face challenges in categorizing products that differ significantly from related categories like probiotics. In addition, unlike probiotics, postbiotics present unique challenges for pharmaceutical‐grade characterization as postbiotic formulations may contain different components (cells, cell fragments, and metabolites), requiring suitable analytical methods for identification and quantification. Further, due to possible systematic absorption of postbiotic compounds, careful consideration regarding their possible immunogenic reactions is required. However, recent developments in achieving the approval of safety assessment for three postbiotic formulations obtained from *Bacteroides xylanisolvens*, *Akkermansia muciniphila*, and *Mycobacterium manresensis* by the EFSA can serve as models for regulatory requirements (Vinderola et al. 2022b). Further, regulatory framework on postbiotic should consider that any product labels include the name of the microorganism (genus, species, and strain) from which the ingredient is derived, the type of postbiotic, quantity in appropriate units guaranteed at end of shelf‐life, serving size, storage conditions, expiry date, and corporate contact details (Guglielmetti 2023; Guglielmetti et al. 2025; Vinderola et al. 2022b). The regulatory framework is evolving toward recognizing postbiotics as preparations of inanimate microorganisms able to confer health benefits. However, international consensus is crucial for establishing consistent evaluation criteria safety requirements and innovations that will facilitate wider global market access. The postbiotic regulatory landscape represents an emerging field requiring coordinated efforts among industry, academia, and regulatory authorities. As the scientific understanding of postbiotics advances, regulatory frameworks must evolve to provide clear pathways for safe and effective products to reach consumers (Guglielmetti 2023; Guglielmetti et al. 2025).

## 8 Conclusions

This review critically evaluates both traditional and emerging nonthermal technologies for postbiotic production, emphasizing their effectiveness, scalability, and industrial applicability. It assesses the potential of nonthermal methods to address challenges in postbiotic development, such as maintaining bioactivity, ensuring safety, and preserving sensory qualities, while also adhering to regulatory standards and consumer expectations. Through a systematic literature review, this study explores whether nonthermal technologies can revolutionize postbiotic production and enhance their integration into functional foods. The findings indicate that nonthermal technologies—such as HPP, PEFs, ultrasound, and cold plasma—offer significant advantages over conventional thermal processes. These methods preserve structural integrity and enhance the bioactive properties of postbiotic components, which are essential for health benefits. Evidence suggests that postbiotics produced through nonthermal methods exhibit superior metabolic and immune‐modulatory effects, support gut barrier function, and hold promise in reducing the risk of chronic diseases, while overcoming the limitations of conventional thermal methods. Nonthermal production methods also align with contemporary functional food manufacturing requirements, enhancing microbial safety, stability, and consistency necessary for clinical applications. These techniques optimize processing efficiency while retaining health‐promoting characteristics and align with industry trends toward clean‐label products, offering both environmental and economic benefits. The integration of nonthermal processing with innovative fermentation strategies can facilitate the creation of tailored postbiotic profiles for specific health needs and consumer preferences. This review also underscores the interdisciplinary nature of postbiotic research, encompassing microbiology, food engineering, biotechnology, and public health. By comparing traditional and nonthermal technologies, it demonstrates how innovative processing methods drive the development of next‐generation functional food ingredients. The findings support the notion that nonthermal technologies represent a fundamental shift in postbiotic production and utilization, further enhanced by their ability to meet regulatory standards. Despite these advancements, several limitations warrant consideration. Although nonthermal methods offer technical advantages, large‐scale validation of their long‐term health impacts remains insufficient. Regulatory frameworks are still evolving, with regional differences posing challenges to harmonization. Optimizing process parameters requires further research to balance effectiveness, safety, and cost‐efficiency during scale‐up while carefully considering removing inconsistencies in study design and reporting standards through development of standardized protocols for production and quality assurance. Innovation is necessary to refine nonthermal technologies with advanced fermentation processes, enabling the precise engineering of postbiotics for targeted applications. Overall, nonthermal postbiotic production faces scientific and practical hurdles but provides promising opportunities for further innovation and development.

## Author Contributions

**Rohit Thirumdas**: conceptualization, writing–review and editing, writing–original draft. **Priti Mudgil**: conceptualization, writing–review and editing, writing–original draft, project administration.

## Conflicts of Interest

The authors declare no conflicts of interest.
